# Transcriptome and Proteome Exploration to Provide a Resource for the Study of *Agrocybe aegerita*

**DOI:** 10.1371/journal.pone.0056686

**Published:** 2013-02-13

**Authors:** Man Wang, Bianli Gu, Jie Huang, Shuai Jiang, Yijie Chen, Yalin Yin, Yongfu Pan, Guojun Yu, Yamu Li, Barry Hon Cheung Wong, Yi Liang, Hui Sun

**Affiliations:** 1 State Key Laboratory of Virology, College of Life Sciences, Wuhan University, Wuhan, People's Republic of China; 2 Department of Clinical Immunology, Guangdong Medical College, Dongguan, People's Republic of China; 3 Key Laboratory of Fermentation Engineering (Ministry of Education), Hubei University of Technology, Wuhan, People's Republic of China; 4 Key Laboratory of Combinatorial Biosynthesis and Drug Discovery (Ministry of Education), Wuhan University, Wuhan, People's Republic of China; 5 Molecular Diagnosis Center, The First Affiliated Hospital of Henan University of Science and Technology, Luoyang, People's Republic of China; University of Sydney, Australia

## Abstract

**Background:**

*Agrocybe aegerita*, the black poplar mushroom, has been highly valued as a functional food for its medicinal and nutritional benefits. Several bioactive extracts from *A. aegerita* have been found to exhibit antitumor and antioxidant activities. However, limited genetic resources for *A. aegerita* have hindered exploration of this species.

**Methodology/Principal Findings:**

To facilitate the research on *A. aegerita*, we established a deep survey of the transcriptome and proteome of this mushroom. We applied high-throughput sequencing technology (Illumina) to sequence *A. aegerita* transcriptomes from mycelium and fruiting body. The raw clean reads were *de novo* assembled into a total of 36,134 expressed sequences tags (ESTs) with an average length of 663 bp. These ESTs were annotated and classified according to Gene Ontology (GO), Clusters of Orthologous Groups (COG), and Kyoto Encyclopedia of Genes and Genomes (KEGG) metabolic pathways. Gene expression profile analysis showed that 18,474 ESTs were differentially expressed, with 10,131 up-regulated in mycelium and 8,343 up-regulated in fruiting body. Putative genes involved in polysaccharide and steroid biosynthesis were identified from *A. aegerita* transcriptome, and these genes were differentially expressed at the two stages of *A. aegerita*. Based on one-dimensional gel electrophoresis (1-DGE) coupled with electrospray ionization liquid chromatography tandem MS (LC-ESI-MS/MS), we identified a total of 309 non-redundant proteins. And many metabolic enzymes involved in glycolysis were identified in the protein database.

**Conclusions/Significance:**

This is the first study on transcriptome and proteome analyses of *A. aegerita*. The data in this study serve as a resource of *A. aegerita* transcripts and proteins, and offer clues to the applications of this mushroom in nutrition, pharmacy and industry.

## Introduction

Mushrooms, so-called higher fungi, are popular foods with high medicinal and nutritional values for a long history [1]. Hundreds of pharmaceutical products from mushrooms have been widely used in the anti-tumor/oxidant research and therapy. For example, lectins isolated from *Agaricus bisporus* [2], *Grifola frondosa* [3], *Pleurotus citrinopileatus* [4] have been reported to possess antitumor activities against human cancer cell lines; polysaccharide-K [5], polysaccharide peptide [6], [7] and lentinan [8] have been used as adjuvants to modern cancer therapy; extracts from *Inonotus obliquus* [9], *Lactarius deterrimus* and *Boletus edulis* [10] exhibited antioxidant activities. Mushrooms are also environment scavengers that can degrade organic waste in forest ecosystems [11]. The degradation process is carried out by the enzymatic activity on biomass including cellulose and lignin, and this process benefits the carbon and nitrogen cycles of the earth [12], [13]. *Agrocybe aegerita*, also called black poplar mushroom, is one of the most cultivated mushrooms in Asia, and has been highly valued as a functional food for its anti-tumor, anti-oxidant, anti-fungal, hypocholesterolemic and hypolipidemic effects [14], [15], [16]. Peroxygenase [17] and haloperoxidase [18] isolated from *A. aegerita* were proved to be promising biocatalysts in biotechnological applications. Though *A. aegerita* has huge medicinal and industrial potentials, the publicly available data are not sufficient for elucidating the molecular mechanisms underlying *A. aegerita* development: only a few EST and nucleotide sequences exist in the GenBank database for *A. aegerita* (<400 entries, August 2012). Therefore, there is an urgent need for the genome/proteome-wide study of this mushroom.

Like other mushrooms, the development of *A. aegerita* exhibits two main distinguished morphologies (mycelium and fruiting body), and fruiting body formation is the most complex developmental process [19]. At this stage, complicated regulation on transcriptional and translational levels leads to the dramatic morphological changes [20]. Transcriptome analysis using high-throughput sequencing and proteome analysis using LC-MS/MS would help us to understand the molecular mechanism underlying development regulation. Transcriptome represents the complete set of transcripts in a cell in the context of a specific developmental stage or a physiological environment [21]. Transcriptome provides information on transcript quantity and gene expression variation. To date, the high-throughput sequencing has been applied to the genome/transcriptome analysis of higher fungi, including *Coprinopsis cinerea* [22], *Laccaria bicolor* [23], [24], *Schizophyllum commune* [25] and *Ganoderma lucidum* [26]. High-throughput sequencing technology (next-generation sequencing technology) has already revolutionized the way we study the transcriptome. The advantages of this technique are the large amounts generated (5–10 Gbp total per run) and high sequencing accuracy [27]. The combination of one-dimensional gel electrophoresis (1-DGE) with mass spectrometry (MS) constitutes one of the main methods for proteomics analysis [28]. Liquid chromatography tandem mass spectrometry (LC-MS/MS) can theoretically identify any protein when the genome sequence of the organism under study is available [29]. Proteomics analysis has been used to identify the proteins of interest from fungi such as *Pleurotus ostreatus* [30], *Pleurotus tuberregium* [31], *Sparassis crispa* and *Hericium erinaceum* [12].

To understand the complexity of the transcriptome during *A. aegerita* development at the genomic level, we performed the first global analysis of the transcriptomes from mycelium and fruiting body of *A. aegerita* using the Illumina paired-end sequencing technology. This comprehensive analysis of the transcriptomes may substantially improve the global view of the potential molecular mechanisms involved in *A. aegerita* development and pave the way for its further analysis. Moreover, one-dimensional gel electrophoresis and mass spectroscopy analysis were used to survey expressed proteins in *A. aegerita*. The transcriptomic and proteomic data supply important information on *A. aegerita*, and provide important clues to the deep exploration of this species.

## Results and Discussion

### Illumina sequencing and *de novo* assembly

To gain a global overview of the *A. aegerita* transcriptome, we sequenced the cDNA samples of mycelium and fruiting body using the Illumina paired-end sequencing technology. Each sample produced over 1 G raw data, from paired-end (PE) reads with a single read length about 75 bp and a Q20 percentage (percentage of sequences with sequencing error rate lower than 1%) over 95% (**Table S1**). These data showed that the quality of sequencing was high enough for further analysis.

After removal of adaptor sequences, duplication sequences, ambiguous reads and low-quality reads, we obtained a total of over 14 million 75 bp sequencing reads generated from the two 200 bp (±25 bp) insert libraries. These short reads were assembled into 81,026 mycelium contigs and 73,303 fruiting body contigs with mean lengths of 233 bp and 246 bp, respectively (**Table 1**). By PE joining and gap filling, the contigs were then assembled into 44,547 scaffolds in mycelium and 38,383 scaffolds in fruiting body with mean lengths of 409 bp and 456 bp, respectively. Scaffolds were further assembled into ESTs, and we obtained 28,086 mycelium ESTs and 25,253 fruiting body ESTs with average lengths of 570 bp and 624 bp, respectively. ESTs from the two libraries were combined, and 36,134 ESTs were finally obtained with a mean length of 663 bp and an N50 of 940 bp, suggesting that the short reads were effectively assembled (**Table 1**).

**Table 1:** General features of the *A. aegerita* transcriptome.

	Mycelium	Fruiting body	Total
Total Nucleotides (bp)	1,116,819,840	1,014,944,484	2,131,764,324
Average read length (bp)	148	148	148
Number of contigs	81,026	73,303	154,329
Average contig length (bp)	233	246	-
Number of scaffolds	44,547	38,383	82,930
Average scaffold length (bp)	409	456	-
Number of ESTs	28,086	25,253	**36,134**
Average EST length (bp)	570	624	**663**
Length of all ESTs (bp)	16,013,419	15,760,105	**23,957,359**
N50 of ESTs (bp)	723	841	**940**

The N50 indicates that 50% of all bases are contained in ESTs at least as long as N50.

**Figure 1:**
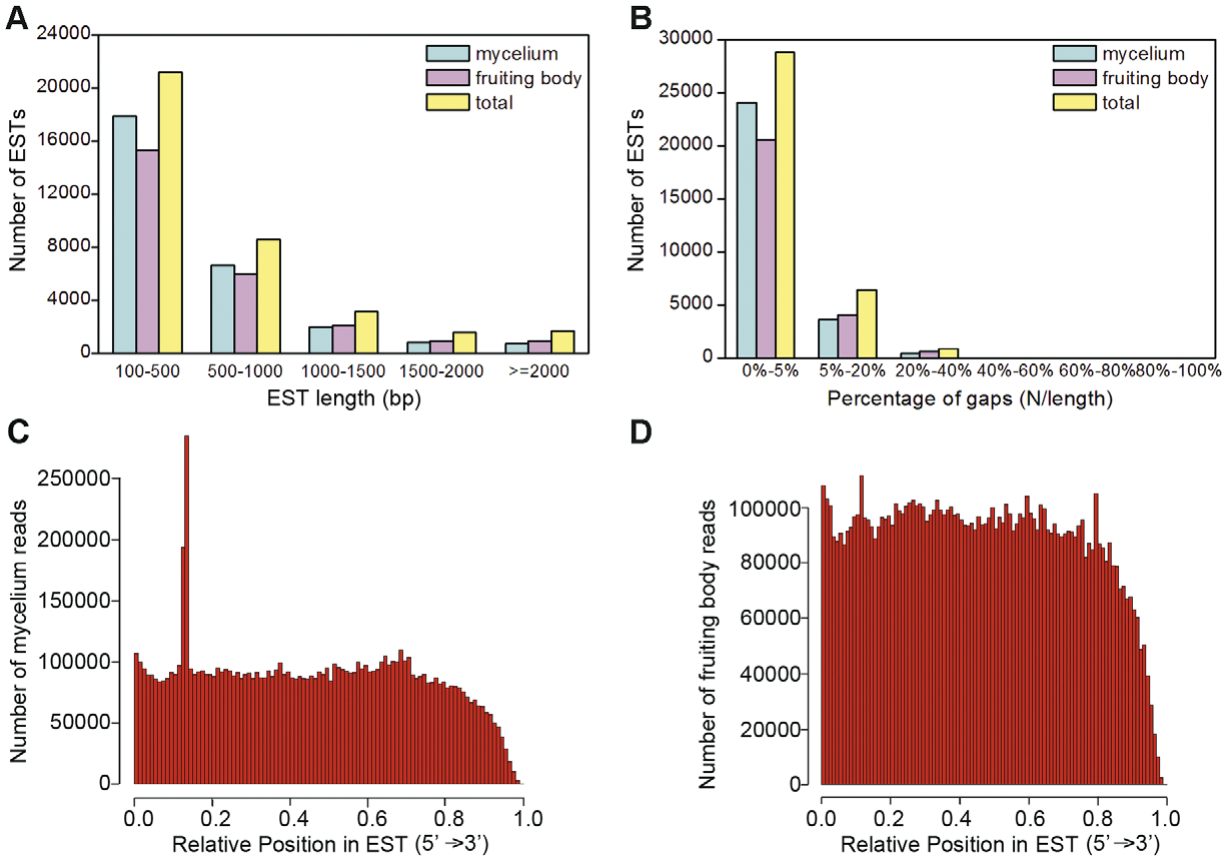
Overview of the assembled sequences in *A. aegerita* transcriptome. (A) Length distribution of assembled ESTs. (B) Gap distribution of ESTs. Gaps represent ratio of uncertain bases (‘N’s) in ESTs. Random distribution of Illumina sequencing reads in the assembled ESTs from mycelium (C) and fruiting body (D). The x-axis indicates the relative position of sequencing reads in the assembled ESTs. The orientation of EST is from 5′ end to 3′ end.

To evaluate the quality of dataset, we further analyzed size, gap and read distributions of the assembled ESTs. The size distribution indicated that the lengths of the majority of ESTs were 100–500 bp, with 17,869 (63.62%) in mycelium, 15,297 (60.58%) in fruiting body, and 21,163 (58.57%) in the total set (**Figure 1A**). Sequence size distributions for mycelium and fruiting body were consistent, implying that the Illumina sequencing solution was reliable and reproducible. Gap (‘N’ amount/sequence length) distribution of these ESTs was shown in **Figure 1B**. The majority of ESTs (85.60% in mycelium, 81.48% in fruiting body and 79.75% in the total set) produced gaps of 0–5%. In addition, sequencing bias was analyzed by detecting random distribution of reads in assembled ESTs (**Figure 1C & D**). The 3′ ends of all assembled ESTs contained relatively fewer numbers of reads, but most positions from the two samples displayed even distribution.

In the absence of a genome assembly it is only possible to make an approximation of transcriptome coverage. The genome sizes of *C. cinerea* [22], *S. commune* [25], *G. lucidum* [26] were 37 Mb, 38.5 Mb and 43.3 Mb, respectively. As a result, we estimated the average genome size of mushrooms to be 40 Mb. In this study, we obtained approximately 2.13 Gbp of sequence data (**Table S1**), representing at least 53-fold coverage. However, higher coverage and genome sequence of *A. aegerita* are required for complete assembly of transcriptomic sequences. Despite the limitation in transcriptome coverage, we identified a large set of *A. aegerita* sequences, which provide a rich source of information for further investigation.

### Function annotation of *A. aegerita* transcriptome

**Figure 2:**
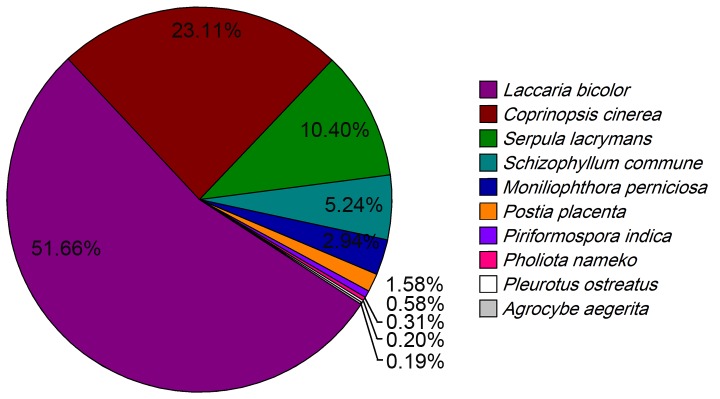
Species distribution of the BLASTx results. The figure shows the species distribution of EST BLASTx results against the NCBI-nr protein database (E<10^−5^) and the proportions for each species. Different colors represent different species, and the top 10 species are shown.

To annotate *A. aegerita* transcriptome, all the ESTs were first searched using BLASTx against the NCBI non-redundant (nr) protein database with a cut-off E-value of 10^−5^. A total of 17,813 ESTs (49.30%) returned an above cut-off BLAST result (**Table S3**). The species distribution of the best match result for each EST was shown in **Figure 2**. Homology analysis indicated that 51.66% of *A. aegerita* ESTs showed the greatest similarity to *Laccaria bicolor*, followed by *Coprinopsis cinerea* (23.11%), *Serpula lacrymans* (10.40%), *Schizophyllum commune* (5.24%) and *Moniliophthora perniciosa* (2.94%). The species distribution suggested that *A. aegerita* had a closer relationship with the model mushroom *L. bicolor*. Due to lack of protein information of *A. aegerita* in nr database, only 0.19% of the ESTs matched with *A. aegerita* sequences.

**Figure 3:**
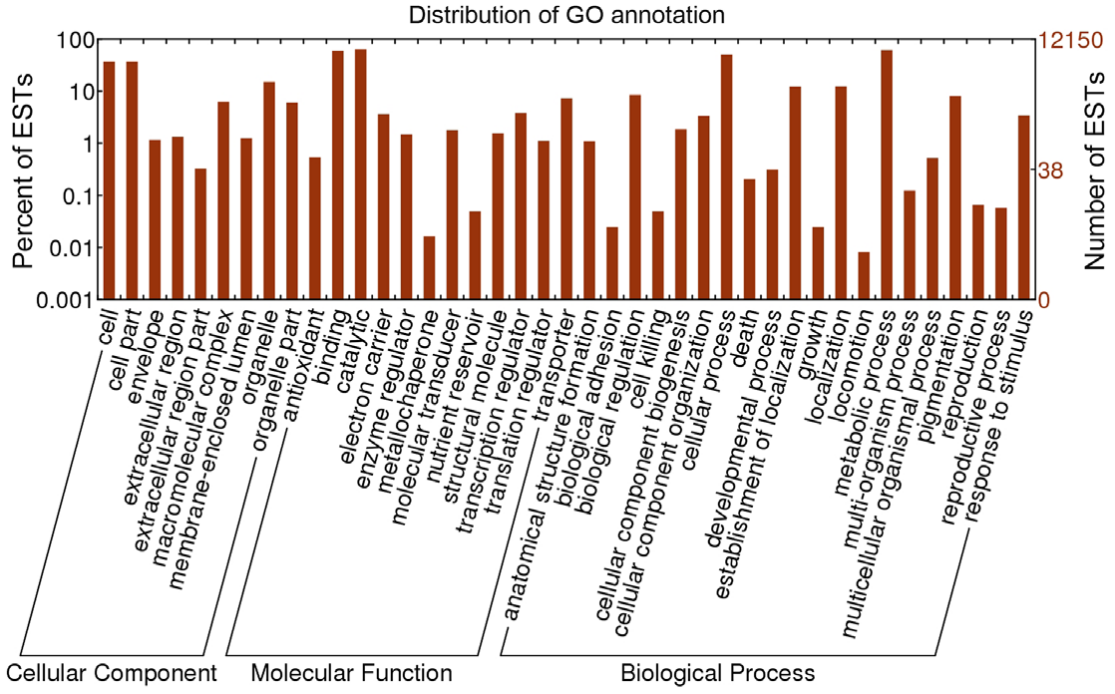
Gene Ontology classification of the *A. aegerita* transcriptome. Histogram presentation of the GO annotation was generated automatically by the web histogram tool WEGO (http://wego.genomics.org.cn/cgi-bin/wego/index.pl) using the newest GO archive provided. The results are summarized in three main GO categories: cellular component, molecular function and biological process. The right y-axis indicates the number of ESTs in a category. The left y-axis indicates the percentage of a specific category of ESTs in that main category. One EST could be annotated into more than one GO term.

**Figure 4:**
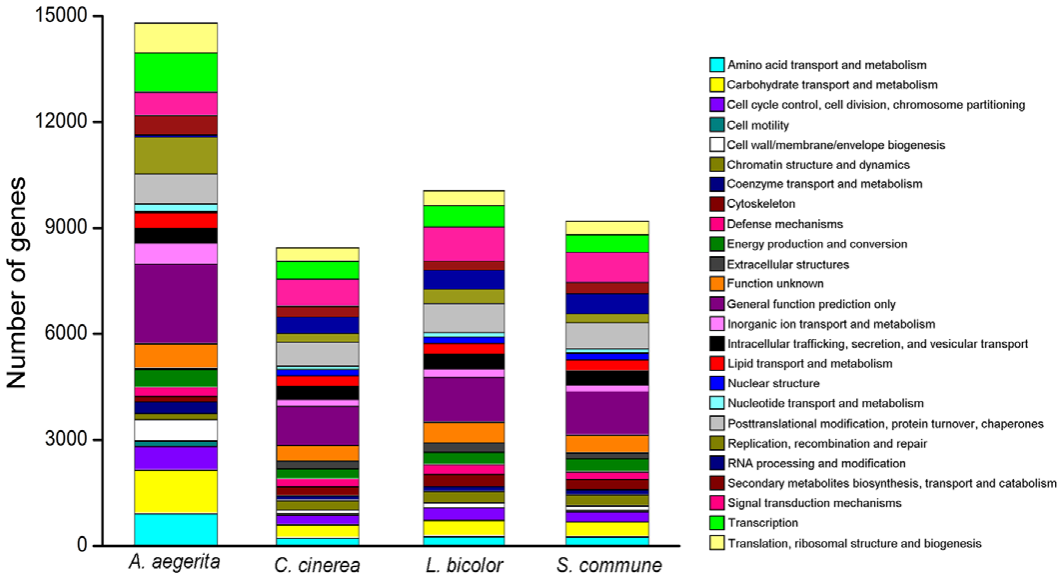
COG functional categories in the four basidiomycete species. Out of 17,813 nr hits, 7,634 *A. aegerita* sequences were assigned to one or more COG functional categories. Different colors are used to indicate the 25 COG functional categories.

**Figure 5:**
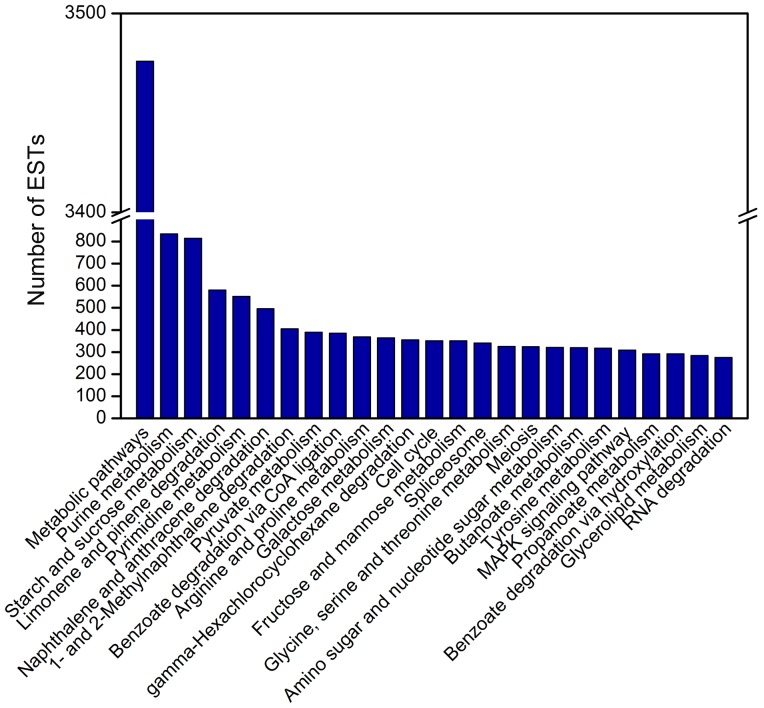
The top 25 KEGG categories of *A. aegerita* ESTs.

1. **Gene Ontology annotation.** Based on nr annotation, Gene Ontology (GO) [32] assignments were used to classify the functions of *A. aegerita* ESTs. Out of the 17,813 most significant BLASTx hits against the nr database, a total of 12,419 ESTs were categorized into 41 functional groups (**Figure 3**). Under each of the three main categories (biological process, cellular component and molecular function) of the GO classification, “metabolic process”, “cell”, “cell part” and “catalytic activity” terms occupied the largest proportion. We also noticed a high percentage of ESTs from categories of “cellular process”, “organelle” and “binding”. Only a few ESTs were categorized to the terms of “locomotion”, “extracellular region part” and “metallochaperone activity” (**Figure 3**). GO annotation also predicted proteins with antioxidant activity in *A. aegerita* [33]. Moreover, the major processes in *A. aegerita* were consistent with those in *L. edodes*, *C. cinerea*, *P. ostreatus* and *L. bicolor* [20].
2. **COG annotation.** To further evaluate the completeness of our transcriptome and the effectiveness of our annotation process, we searched the annotated sequences for the genes involved in COG classifications [34]. COG-annotated putative proteins were classified functionally into at least 25 molecular families, such as Nuclear structure, Cytoskeleton, Extracellular structures and Energy production and conversion. A total of 7,634 *A. aegerita* ESTs were divided into one or more COG functional categories. We also compared the COG categories of *A. aegerita* with three other mushrooms including *C. cinerea*, *L. bicolor* and *S. commune* (http://www.jgi.doe.gov/) (**Figure 4**). *A. aegerita* and these mushrooms were similar in each COG category. However, *A. aegerita* had more ESTs in most categories, especially in Defense metabolisms, Carbohydrate transport and metabolism and Secondary metabolites biosynthesis, transport and catabolism, but fewer ESTs for Extracellular structures, RNA processing and modification and Nuclear structure.
3. **KEGG annotation.** To identify the biological pathways in *A. aegerita*, we mapped the sequences to the reference canonical pathways in KEGG. And a total of 10,246 ESTs were mapped into 150 KEGG pathways. The top 25 KEGG metabolic pathways were shown in **Figure 5**. Highest numbers of ESTs were classified under metabolic pathways (3,476, 33.9%), followed by purine metabolism (834, 3.44%), starch and sucrose metabolism (814, 3.35%) and limonene and pinene degradation (580, 2.39%). The other highly represented pathways included pyrimidine metabolism (551, 2.27%), naphthalene and anthracene degradation (496, 2.04%). Function annotations of *A. aegerita* transcriptome (**Figure 4** **&** **5**) showed that carbohydrate and amino acid metabolisms were active in this mushroom, suggesting that *A. aegerita* was a nutritious food source containing various carbohydrates and proteins as other mushrooms [35].
4. **Carbohydrate and purine metabolisms in** ***A. aegerita*** **.** Similar to KEGG mapping of *A. aegerita*, a large number of genes were involved in starch and sucrose metabolism and purine metabolism in *L. bicolor* and *S. commune* (**Figure S2**). To further analyze the two important metabolic pathways, we identified some putative ESTs encoding the enzymes involved in the two pathways (**Table 2**). In purine metabolism, both amidophosphoribosyltransferase (EC 2.4.2.14) [36] and adenylosuccinate lyase (EC 4.3.2.2) [37] play a critical role in cellular replication and metabolism. And we identified two amidophosphoribosyltransferases and three adenylosuccinate lyases from *A. aegerita* transcriptome. In starch and sucrose metabolism, we identified three endoglucanases (EC 3.2.1.4) from *A. aegerita* transcriptome and these enzymes play an important role in cellulose degradation [38]. Trehalose is a non-reducing disaccharide that is found in animals, fungi and plants [39]. Trehalose phosphatase (EC 3.1.3.12) was reported to be involved in trehalose biosynthesis [40] and the enzyme has regulatory roles in sugar metabolism, carbon storage, growth and development [41]. In this study, two ESTs encoding trehalose phosphatases were found. Four glycogen synthases (EC 2.4.1.11) that play an important role in the glucose cycle [42] were also identified. The updated KEGG reference maps of the two metabolic processes, with reference to the ESTs identified in *A. aegerita* transcriptome, were shown in **Figure S3** and **Figure S4**. Genes involved in most steps of each metabolic pathway were discovered in *A. aegerita* transcriptome.
5. **Secondary metabolism.** Many fungi produce secondary metabolites that confer selective advantages in nature by protecting against predators and assisting in the acquisition of nutrients [43]. Most of fungal metabolites had antibacterial, antifungal or antitumor activity [44]. The biosynthesis of a large number of natural products requires the participation of sophisticated molecular mechanisms known as polyketide synthases (PKS) and nonribosomal peptide synthases (NRPS) [45]. Examination of *A. aegerita* transcriptome suggested potential for production of an array of bioactive compounds. Some putative PKSs and NRPSs were identified in *A. aegerita* transcriptome (**Table S4**). A minimum of 11 putative genes in *A. aegerita* encoded PKSs responsible for biosynthesis of polyketides. Polyketides are the most abundant fungal secondary metabolites and are widely used as antimicrobial, antifungal, immunosuppressant and anticancer agents [44], [46]. The biosynthesis of polyketides also involved the activity of cytochrome P450-like enzymes. The cytochrome P450 monooxygenases comprise an ancient family of enzymes that oxidize a wide range of targets and are involved in various metabolic pathways [47]. A total of 257 CYP sequences representing 29 CYP families [48] were observed in *A. aegerita* transcriptome (**Table S5**). Highest number of *A. aegerita* CYPs (62 members) belonged to family CYP5144 and some members of this family are known to be involved in xenobiotic metabolism [49]. *Aspergillus flavus*, *A. oryzae*, *A. niger* and *Fusarium verticillioides*, all of which are known for their capability of producing various secondary metabolites, have the largest numbers of CYP5144 members [50]. The presence of a large number of CYP5144 members in *A. aegerita* suggested that abundant secondary metabolites were produced in this mushroom. Five *A. aegerita* CYPs belonged to family CYP530. CYP530 family seems to be specific to fungi and participates in degradation of various fatty acid and hydrocarbons, allowing fungi to utilize these materials as nutrient sources [50]. Moreover, *A. aegerita* had a higher number of P450 genes than *G. lucidum* (197 genes), *Postia placenta* (186 genes) and *Phanerochaete chrysosporium* (148 genes) [26]. We also identified 12 non-ribosomal peptide synthases that might yield cyclic peptide antibiotics (**Table S4**).

**Table 2:** Number of annotated unique sequences involved in purine and carbohydrate metabolisms.

Enzyme code	Enzyme name	Number of unique ESTs
**purine metabolism**		
3.5.3.19	Ureidoglycolate hydrolase	1
3.5.1.5	Urease	2
2.7.7.48	RNA-directed RNA polymerase	3
2.7.6.1	Ribose-phosphate pyrophosphokinase	5
2.7.1.40	Pyruvate kinase	2
1.7.3.3	Urate oxidase	3
2.4.2.14	Amidophosphoribosyltransferase	2
2.1.2.2	Phosphoribosylglycinamide formyltransferase	1
6.3.5.3	Phosphoribosylformylglycinamidine synthase	6
6.3.2.6	Phosphoribosylaminoimidazole-succinocarboxamide synthase	2
4.1.1.21	Phosphoribosylaminoimidazole carboxylase	4
3.5.4.3	Guanine deaminase	3
3.6.1.11	Exopolyphosphatase	1
3.5.3.4	Allantoicase	1
4.3.2.2	Adenylosuccinate lyase	3
2.7.4.3	Adenylate kinase	1
4.6.1.1	Adenylate cyclise	3
2.7.1.20	Adenosine kinase	1
2.4.2.7	Adenine phosphoribosyltransferase	1
3.1.3.5	5 –nucleotidase	3
**Starch and sucrose metabolism**		
5.3.1.9	Glucose-6-phosphate isomerise	5
3.2.1.91	Cellulose -beta-cellobiosidase	3
3.2.1.4	Endoglucanase	3
3.2.1.21	Beta-glucosidase	2
3.2.1.1	Alpha-amylase	3
2.4.1.11	Glycogen synthase	4
2.7.1.1	Hexokinase	2
3.6.1.9	Nucleotide pyrophosphatase	1
5.4.2.2	Phosphoglucomutase	3
3.1.3.12	Trehalose phosphatase	2
1.1.1.22	UDP-glucose 6-dehydrogenase	1
2.7.7.9	UTP-glucose-1-phosphate uridylyltransferase	4

In the present study, we found some genes that might be involved in the biosynthesis of PKs and NRPs in *A. aegerita*. And these compounds have not previously been isolated from this mushroom. This example shows that transcriptome analyses can provide insight into the complete chemical profile of an organism. Our findings suggested that *A. aegerita* contained abundant secondary metabolites and some of the biosynthesis pathways might contribute to the medicinal values of this species. The KEGG pathways identified in this study established a foundation for further research on metabolic pathways in *A. aegerita*.

### Analysis of differentially expressed genes (DEG)

**Figure 6:**
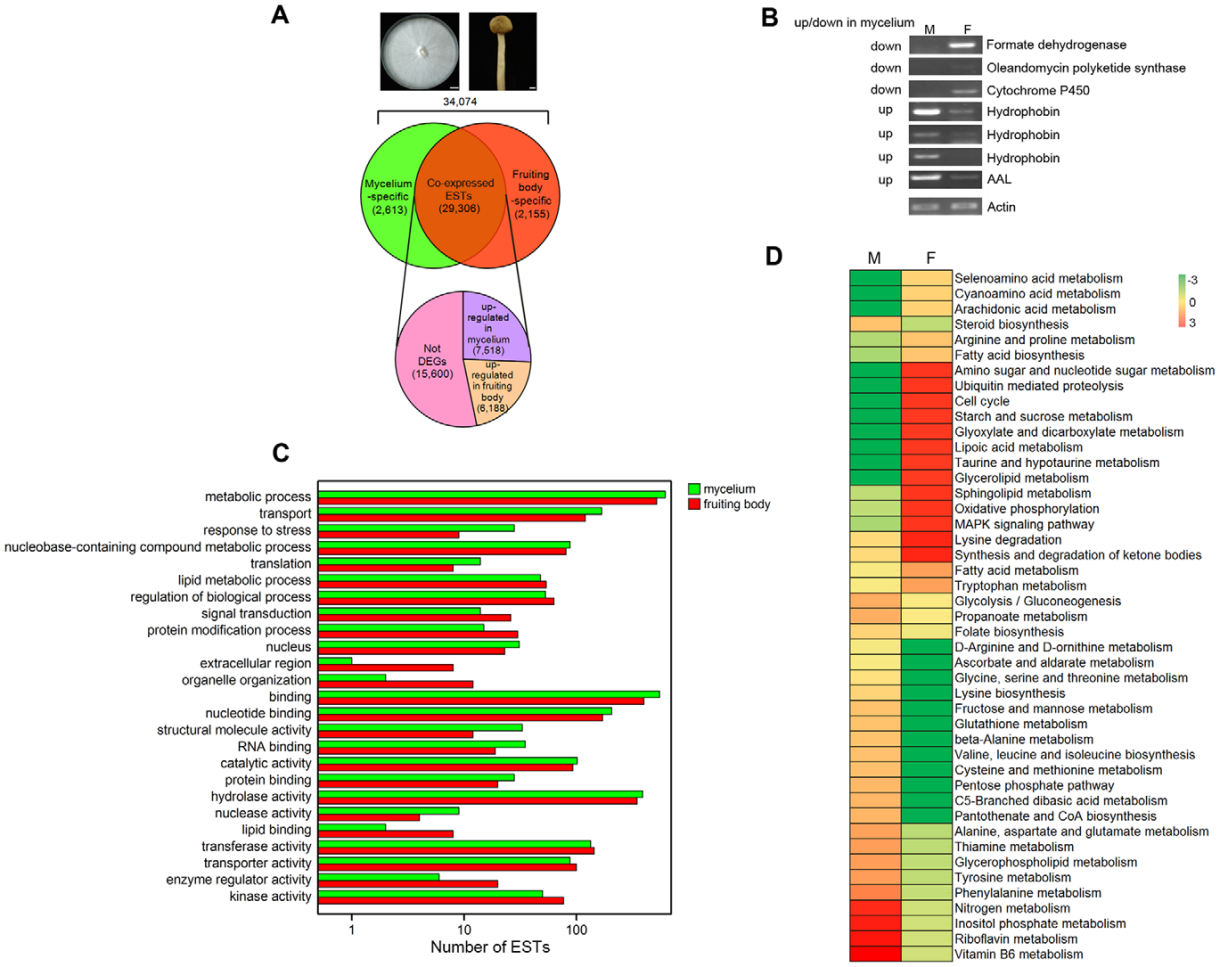
Function categorization of DEGs. (A) Venn diagrams of differentially expressed genes from two developmental stages of *A. aegerita*. Representative pictures of vegetative mycelium at 14 days (left) and fruiting body (right) are shown; Scale bars, 1 cm. (B) Validation of gene expression by semi-quantitative RT-PCR. M: mycelium; F: fruiting body. The captions at the left of the gel pictures indicate the predicted up or down-regulated expression of ESTs in mycelium vs. fruiting body by the reads abundance of Illumina sequencing. The right of gel pictures shows the putative encoding protein of each gene. At least three rounds of independent replication were used for each primer pair. The putative *actin* gene (*AA_19720*) was used as a reference. (C) The top 25 represented GO annotations of differentially expressed genes. Crossbands in green represent up-regulated ESTs in mycelium, and crossbands in red represent up-regulated ESTs in fruiting body. (D) KEGG annotation of DEGs. The heatmap shows the pathway annotations of DEGs between mycelium (denoted as M) and fruiting body (denoted as F). Each row represents a differentially expressed metabolic pathway. The color scale indicates expression levels with red representing up-regulated expression and green representing down-regulated expression.

The RPKM method (Reads Per kb per Million reads), first described by Mortazavi et al. in 2008 [51], can eliminate the influence of sequencing bias and different gene lengths, and this method is effective and accurate for the calculation of gene expression. In this study, we used the absolute value of log_2_Ratio>1 and FDR≤0.001 as the threshold to determine the differentially expressed genes between the two developmental stages [52]. We identified a total of 18,474 differentially expressed ESTs at the two stages (**Table S6**), with 2,613 and 2,155 ESTs specifically expressed in mycelium and fruiting body, respectively (**Figure 6A**). 7,518 ESTs were up-regulated in mycelium and 6,188 ESTs were up-regulated in fruiting body amongst 29,306 co-expressed ESTs at the two stages (**Figure 6A**). The log_2_ratio fold changes between the two stages were from −27 to 27 (**Table S6**). The major (2,734) of up-regulated ESTs in mycelium showed changes in expression between one and two fold (**Figure S5**). The top ten differentially expressed ESTs at the two developmental stages have been listed in **Table S7**. The most significant expression difference was for *AA_21574*, annotated as Aa1-Pri4, which was 27 fold higher in mycelium than in fruiting body. The expression pattern of Aa1-Pri4 in this study was consistent with the previous report [53].

**Figure 7:**
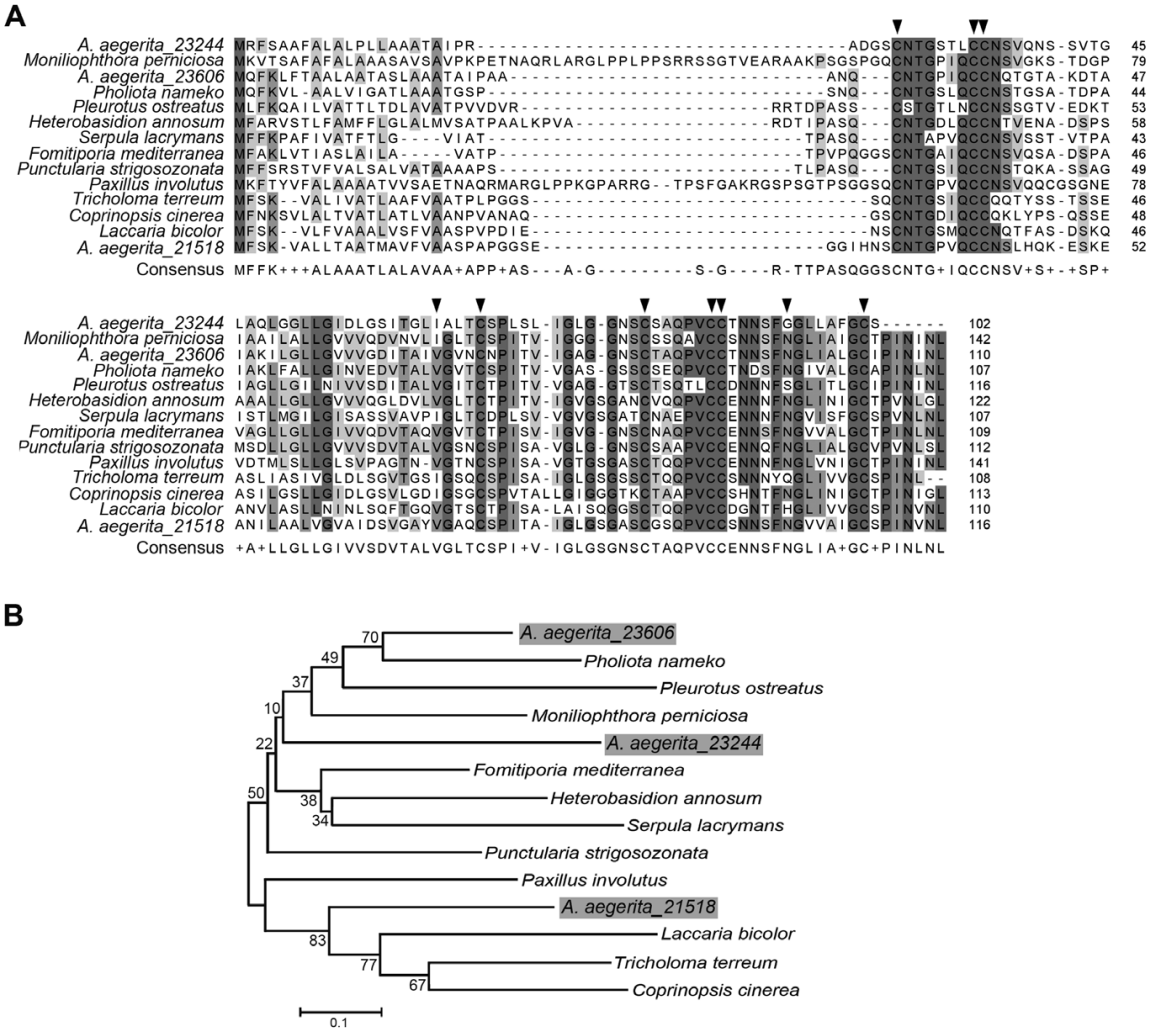
Comparison between *A. aegerita* hydrophobins and other fungi. (A) Alignment of hydrophobin sequences from fungi. The hydrophobin protein sequences of *P. nameko*, *P. ostreatus*, *M. perniciosa*, *P. strigosozonata*, *F. mediterranea*, *H. annosum*, *S. lacrymans*, *P. involutus*, *L. bicolor*, *T. terreum* and *C. cinerea* are available under GenBank accession numbers BAB84547.1, CAB41405.1, XP_002389333.1, EIN08599.1, EJD00400.1, ABA46362.1, EGO28137.1, AAX51848.1, XP_001885703.1, AAL05426.1 and XP_001831661.1. Strictly conserved residues are indicated by grey shadows; black arrows represent cysteine residues of the consensus sequence. (B) Phylogenetic analysis of the putative hydrophobins of *A. aegerita* with above-cited sequences. Phylogenetic analysis was performed with MEGA 4.0 [112]. The evolutionary history was inferred using the Neighbor-Joining method. The bootstrap consensus tree inferred from 1000 replicates was taken to represent the evolutionary history of the taxa analyzed.

**Figure 8:**
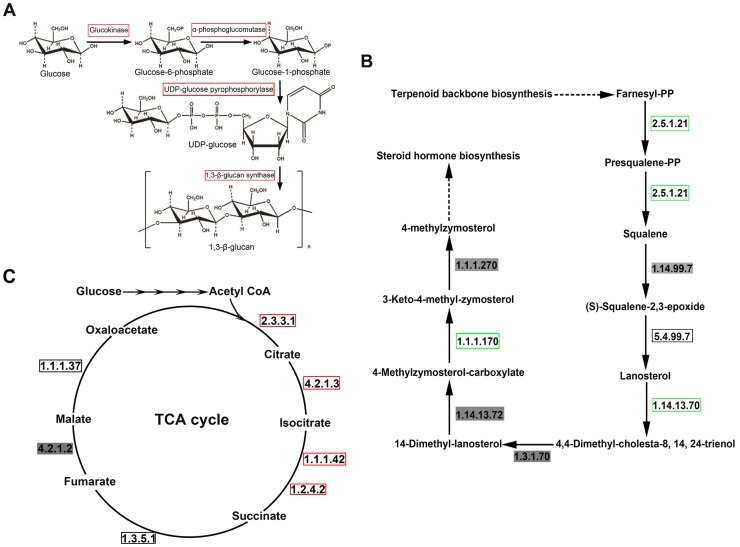
Putative components of polysaccharide biosynthesis, steroid biosynthesis and TCA cycle. (A) The polysaccharide biosynthesis pathway. (B) The steroid biosynthesis pathway. (C) Pathway map of TCA cycle. Green boxes represent genes up-regulated in mycelium; red boxes represent genes up-regulated in fruiting body; black boxes represent genes having common expression levels at the two stages; words in a grey shadow represent that no genes were identified in the process from *A. aegerita* transcriptome.

1. **Validation of the DEG results by RT-PCR analysis.** To validate gene expression profiles, we conducted semi-quantitative RT-PCR to confirm the expression levels of 7 selected ESTs (**Figure 6B**). These ESTs exhibited high abundance and were differentially expressed between mycelium and fruiting body. *AA_19720* was the *actin* gene of *A. aegerita* and used as the reference. All these ESTs were amplified successfully and resulted into a single band of the expected size (158 bp to 282 bp approximately). The results showed that four ESTs exhibited higher expression level in mycelium, including genes encoding hydrophobins (*AA_33178*, *AA_34533* and *AA_34539*) and *A. aegerita* lectin (*AAL*, *AA_12497*) while the others were up-regulated in fruiting body, including genes encoding formate dehydrogenase (*AA_10799*), polyketide synthase (*AA_10787*) and cytochrome P450 (*AA_36106*). The expression patterns of these ESTs were consistent with the reads abundance of Illumina sequencing, suggesting that the DEG analysis was reliable. Both hydrophobins and lectins are cellular structural components and these proteins are related to morphogenesis [54]. Hydrophobin-encoding genes that were developmentally regulated have been isolated from some mushrooms, such as *L. bicolor* [55] and *L. edodes* [56]. Various lectins have been found to have developmental stage-specific expression. For example, lectins from *Pleurotus cornucopiae* [57] and *L. edodes* [54] were abundantly expressed in mycelium, thus revealing their crucial roles in this stage. Lectins seem to be involved in the formation of hyphal aggregate to stimulate mushroom development [58]. And lectins might cooperate with hydrophobins to maintain cellular structure and promote the development of mushrooms.
2. **GO and KEGG annotations of DEGs.** The GO categories of differentially expressed ESTs revealed that “lipid metabolic process”, “signal transduction”, “protein modification process”, “lipid binding”, “enzyme regulator activity” and “kinase activity” were up-regulated in fruiting body compared with mycelium, indicating these metabolic activities were required for fruiting body development. In mycelium, more ESTs were involved in “transport”, “translation”, “binding”, “structural molecule activity”, “nuclease activity”. “Response to stress” suggested that extra stimulus existed during mycelium growth and development (**Figure 6C**). The log (base 10) of the RPKM values were used to compare 45 significantly different KEGG pathways from both developmental stages (**Figure 6D**). Pathway analysis showed that amino sugar and nucleotide sugar metabolism, ubiquitin mediated proteolysis, cell cycle, starch and sucrose metabolism were more active in the fruiting body than in mycelium. Nitrogen metabolism, inositol phosphate metabolism, riboflavin metabolism and vitamin B6 metabolism were up-regulated in mycelium. Some other similar metabolism pathways were active at both stages, such as carbohydrate and amino acid metabolic pathways. Up-regulated carbohydrate metabolic processes in mycelium included ESTs involved in: fructose and mannose metabolism and pentose phosphate pathway. Several carbohydrate metabolic pathways such as starch and sucrose metabolism and glyoxylate and dicarboxylate metabolism were up-regulated in fruiting body. Amino acid metabolic pathways such as glycine, serine, threonine, valine metabolisms were more active in mycelium while arginine, proline and tryptophan metabolisms were more significant in fruiting body. This finding demonstrated the importance of the two metabolic pathways during *A. aegerita* development.
  Previous study on gene expression profiles of *S. commune* indicated that more genes were involved in protein and energy production in mycelium while genes involved in fatty acid metabolism were up-regulated in fruiting body [25]. The DEG analysis of *A. aegerita* indicated that energy production such as glycolysis/gluconeogenesis and pentose phosphate pathway was up-regulated in mycelium. However, energy metabolisms such as nitrogen metabolism and oxidative phosphorylation were also up-regulated in the fruiting body. The up-regulation of energy production at both stages might be required for *A. aegerita* development. The up-regulation of fatty acid biosynthesis and metabolism in the fruiting body was consistent with the changes in *S. commune*. An overall view of the metabolic processes revealed in this study suggested that fruiting body genes were mainly involved in: (1) carbohydrate metabolism, (2) protein degradation, (3) protein production and modification, (3) signal transduction, (4) fatty acid biosynthesis and metabolism, (5) energy production. And the differentially expressed genes provided valuable information on the process of fruiting body development. The formation of fruiting body might be initiated by extra stimulus that activated specific signal transduction, which caused the transition of mycelium to fruiting body by inducing the expression of certain genes. These genes products then regulated fruiting body development by reconstruction of proteome. The reconstruction of proteome occurred via protein degradation and new protein synthesis and modification.
3. **Differentially expressed genes at the two stages.** Based on the DEG analysis, we found great differences in the gene expression profiles of mycelium and fruiting body. Some genes were specifically expressed in mycelium, including transcription factor, serine protease, aromatic peroxygenase, hydrophobin and metalloprotease (**Table 3**). Transcription factors have been identified in some mushrooms such as *C. cinerea* [22] and *S. commune* [25]. In *S. commune*, numerous transcription factors were found to be differentially expressed between development stages, suggesting that transcription factors were important developmental controls. Studies focusing on the phenotypes of knockdown or knockouts of these genes now can be undertaken to explore whether they play important roles in mushroom development. The *A. bisporus* serine proteinase plays an important role in both mycelial nutrition and senescence of the fruiting body [59]. However, serine protease seemed to be more significant for mycelial nutrition in *A. aegerita*. The aromatic peroxygenases (APO) have been discovered in agaric basidiomycetes such as *A. aegerita* and *Coprinellus radians* [60]. APO may be involved in the degradation and detoxification of organic materials [61]. Metalloproteinase members have been found in *P. ostreatus* [62] and *Lepista nuda* [63]. It has been reported that the RNA level of metalloproteinase was abundant at primordial and fruiting body stages in *P. ostreatus* [62]. Metalloproteinase was up-regulated at mycelium stage of *A. aegerita*, suggesting that this enzyme also played a critical role in this stage. Hydrophobins are unique to the fungal kingdom [64] and these proteins are essential for fruiting development in mushrooms [65]. Hydrophobins are small secreted, moderately hydrophobic, self-assembling polypeptides with a conserved distribution of eight cysteine residues that are crucial for proper protein folding [66]. As described for other fungi, hydrophobins were highly expressed in mycelium [54], [67], [68], suggesting that hydrophobins were required to initiate morphological changes in mushrooms. We identified some putative hydrophobins from *A. aegerita* transcriptome, which were also up-regulated in mycelium (**Table S6**). Among the identified hydrophobins, *AA_23606*, *AA_23244* and *AA_21518* showed high identity with hydrophobins from *Pholiota nameko* [GenBank: BAB84547], *P. nameko* [GenBank: BAB84546] and *Tricholoma terreum* [GenBank: AAL05426], respectively. The hydrophobins of *A. aegerita* possessed the eight conserved cysteines and could be aligned with hydrophobins of other fungi (**Figure 7A**). By phylogenetic analysis, we found that *A. aegerita* hydrophobins were phylogenetically similar to hydrophobins of *P. nameko*, *M. perniciosa* and *L. bicolor* (**Figure 7B**). Overall, the DEG results implied the importance of these proteins at the stage of mycelium.
  Some genes such as cytochrome P450, carbohydrate-active enzymes (CAZy enzymes), alcohol dehydrogenase and manganese peroxidase were specifically expressed in fruiting body (**Table 3**). Cytochrome P450 may be involved in the stipe elongation of the fruiting bodies in *L. edodes* [69] and thus showed a higher expression level in fruiting body. Glycoside hydrolase (GH) family members are involved in the degradation of cell wall polysaccharides [70], and these enzymes have been identified in other mushrooms [22], [23], [26]. The up-regulation of GH family members indicated the abundance of these enzymes in fruiting body. Alcohol dehydrogenase was involved in the conversion of carbohydrate into ethanol and showed higher expression in fruiting body which was in accordance with previous observations [69], [71]. Manganese peroxidase was known to catalyze the initial depolymerization of lignin [72]. The presence of this enzyme suggested the wood degrading activity of *A. aegerita*. We also noticed that some genes were commonly expressed at the two stages, such as proteasome regulatory subunit, ribosome protein, sterol dehydrogenase. Proteasome regulatory subunit mainly participates in protein degradation and was reported to be up-regulated in primordium of *Flammulina velutipes* [73]. Both proteasome regulatory subunit and ribosome proteins were highly expressed from mycelium to fruiting body, which implied that an increase in protein varieties was needed for cell differentiation during development. Sterol dehydrogenase was involved in steroid biosynthesis, and steroid has been considered as a bioactive compound from *A. aegerita* [14]. The expression results in this study were suggestive of their involvement in *A. aegerita* development and they should be considered as candidate genes for further studies on the development of this mushroom.
4. **The polysaccharides biosynthesis pathway.** Mushrooms are an important source of polysaccharides with antitumor and immunomodulating activities [74]. The water-soluble 1,3-β-glucans and 1,6-β-glucans are the most active as immunomodulatory compounds among the polysaccharides [75], [76]. To deeply understand polysaccharides in *A. aegerita*, we analyzed the biosynthesis pathway of these glucans. UDP-glucose was the precursor of these glucans, and its biosynthesis involved glucokinase, α-phosphoglucomutase, and UDP-glucose-1-phosphate uridylyltransferase (**Figure 8A**). Genes encoding these enzymes were identified in *A. aegerita* transcriptome. We also identified five 1,3-β-glucan synthases from *A. aegerita* transcriptome and these enzymes play an important role in 1,3-β-glucans biosynthesis [77] (**Table 4**). Analysis of gene expression indicated that genes involved in this pathway were up-regulated in fruiting body, which were consistent with the expression patterns of these genes in *G. lucidum* [26] (**Figure 8A**). Genes involved in this pathway might have stage-dependent expression patterns in mushrooms. In addition, gene encoding the β-glucan biosynthesis-associated protein KRE6 was also discovered in *A. aegerita* transcriptome. And this protein played key roles in the biosynthesis of 1,6-β-glucans [78]. Finally, we also identified some genes (**Table 4**) that were similar to those in other mushrooms, which regulate 1,3-β-glucan and 1,6-β-glucan biosynthesis and play important roles in regulating the polysaccharide content in the cell wall [79], [80].
5. **The steroid biosynthesis pathway.** Steroid and triterpenoid are also bioactive compounds in mushrooms [81]. We further explored the biosynthesis of steroid and triterpenoid in *A. aegerita*. The steroid biosynthesis pathway was shown in **Figure 8B**. Briefly, the first process involves the conversion of farnesyl pyrophosphate (FPP) to squalene catalyzed by squalene synthase (EC 2.5.1.21). Two subsequent enzymatic reactions result in the production of lanosterol, which basically represents the structure of all steroids [82]. In this pathway, two sterol-C4-methyl oxidases and three lanosterol 14-α-demethylases were identified in *A. aegerita*. Lanosterol synthase (EC 5.4.99.7), hydroxysteroid dehydrogenase, squalene synthase (EC 2.5.1.21) and C-3 sterol dehydrogenase were also discovered in the transcriptome (**Table 5**). The previous function analysis of CYPs from *P. chrysosporium* [83] and *P. placenta* [49] showed that enzymes from the CYP512 and CYP5144 families were most likely involved in steroid modification in the two species. In *A. aegerita*, we found 14 genes from the CYP512 family and 62 genes from the CYP5144 family (**Table S5**). However, the exact roles of these CYPs identified in *A. aegerita* require further investigation. Furthermore, the expression profiles of genes involved in this pathway were analyzed. Squalene synthase (EC 2.5.1.21), sterol 14-demethylase (EC 1.14.13.70) and sterol dehydrogenase (EC 1.1.1.170) showed increased expression in mycelium, demonstrating that the steroid biosynthesis was more active at this stage (**Figure 8B**). Fungi were the rich source of sterol, which provided characteristic functions that were necessary for vegetative growth [84]. The active steroid biosynthesis in *A. aegerita* seemed to be required for mycelium growth. Sterols are the major component of membranes, where they regulate permeability and may serve as precursors to steroid hormones involved in the sexual reproduction of mushrooms [85].
  In the terpenoid biosynthesis, several important enzymes encoding genes were identified from the transcriptome such as farnesyl diphosphate synthase, squalene synthase and lanosterol synthase (**Table 5**). In this pathway, farnesyl diphosphate synthase catalyzes the conversion of geranyl pyrophosphates (GPPs) to FPPs which are then converted into lanosterol by squalene synthase and lanosterol synthase. Finally, the lanosterol is metabolized into triterpenoid [86].
6. **TCA cycle.** The tricarboxylic acid (TCA) cycle is one of the iconic pathways in metabolism and is commonly thought of in terms of energy metabolism [87]. In TCA cycle, citrate synthase (EC 2.3.3.1) and aconitate hydratase (EC 4.2.1.3) are involved in the glyoxylate shunt while malate dehydrogenase (EC 1.1.1.37) is involved in the gluconeogenesis [88]. The TCA cycle can be divided into six main processes (**Figure 8C**). Citrate synthase (EC 2.3.3.1) catalyzes the first step in TCA cycle in which oxaloacetate and acetyl-CoA are condensed to generate citrate and CoA. The next step is carried out by aconitase (EC 4.2.1.3), which catalyzes the conversion of citrate to isocitrate. Isocitrate dehydrogenase (EC 1.1.1.42) and oxoglutarate dehydrogenase (EC 1.2.4.2) are involved in the conversion of isocitrate to succinate, which is then converted into fumarate by aconitate dehydrogenase (EC 1.3.5.1). Fumarase (EC 4.2.1.2) then catalyzes the generation of malate from fumarate. At the end of TCA cycle, malate dehydrogenase (EC 1.1.1.37) catalyzes the regeneration of oxaloacetate from malate, and thus the cycle continues [89]. Almost all components in this pathway were present in *A. aegerita* transcriptome, except for fumarase (EC 4.2.1.2) (**Table S8**). DEG analysis suggested that TCA cycle was more active in fruiting body (**Figure 8C**). Transcriptional regulation of TCA pathways in different stages of the fungus *Tuber melanosporum* was also investigated [90]. In the TCA cycle of *T. melanosporum*, aconitate hydratase (EC 4.2.1.3) was up-regulated in mycelium while succinate dehydrogenase (EC 1.3.5.1) was up-regulated in fruiting body. In contrast, aconitate hydratase was up-regulated in fruiting body while succinate dehydrogenase was commonly expressed at both stages of *A. aegerita*. Distinct mechanisms seemed to be responsible for the different regulations of TCA cycle in *A. aegerita* and *T. melanosporum*.

**Table 3:** A list of differentially expressed genes at the two stages.

	EST ID	M-RPKM	F-RPKM	Accession no.	Gene name	Organism
M-specific	AA_11396	8.52	0	ABE60664	multicopper oxidase	*P. chrysosporium*
	AA_15626	5.56	0	XP_001835316	transcription factor	*C. cinerea*
	AA_16353	15.15	0	XP_002911316	aryl-alcohol dehydrogenase	*C. cinerea*
	AA_18723	28.2	0	CAC83024	serine protease	*A. bisporus*
	AA_20098	405.18	0	B9W4V6	aromatic peroxygenase precursor	*A. aegerita*
	AA_21518	410.19	0	AAL05426	hydrophobin	*T. terreum*
	AA_12478	12,32	0	XP_001882073	metalloprotease	*L. bicolor*
F-specific	AA_33566	0	76.85	ACB69807	cytochrome p450	*H. annosum*
	AA_35281	0	226.64	XP_002389285	glycoside hydrolase family 13	*M. perniciosa*
	AA_27472	0	220.42	XP_001835171	glucan 1,3-beta-glucosidase	*C. cinerea*
	AA_35909	0	144.61	XP_001885903	glycoside hydrolase family 61	*L. bicolor*
	AA_31332	0	37.4	XP_001887550	glycosyltransferase family 2	*L. bicolor*
	AA_26107	0	12.88	EGO31002	glycosyltransferase family 22	*S. lacrymans*
	AA_27913	0	18.95	AAZ14917	homeodomain mating-type protein	*C. disseminatus*
	AA_26146	0	22.28	XP_001838093	Hydrolase	*C. cinerea*
	AA_33048	0	22.13	XP_001880244	glycosyltransferase family 69	*L. bicolor*
	AA_26823	0	5.27	XP_001885677	Alcohol dehydrogenase	*L. bicolor*
	AA_25552	0	5.13	ADW41627	manganese peroxidase	*A. praecox*
Common	AA_8188	102.9	51.45	XP_002394087	26s proteasome regulatory subunit	*M. perniciosa*
	AA_3602	215.14	107.52	XP_001831623	60s ribosomal protein L30	*C. cinerea*
	AA_6554	125.01	62.44	XP_001835665	sterol dehydrogenase	*C. cinerea*
	AA_6913	79.83	39.79	BAL02929	ABC transporter-like protein	*P. nameko*
	AA_8800	61.65	30.65	XP_001831326	lipid phosphate phosphatase 1	*C. cinerea*

**Table 4:** Genes involved in polysaccharide biosynthesis and its regulation.

EST ID	Gene name	E-Value	Accession no.	Organism
AA_4781	Glucokinase	2.7E-82	XP_001880386	*L. bicolor*
AA_10967	phosphoglucomutase	0	XP_001875148	*L. bicolor*
AA_32419	UDP-glucose-1-phosphate uridylyltransferase	4.39E-162	XP_001880365	*L. bicolor*
AA_3476	1,3-beta-glucan synthase	0	XP_001878782	*L. bicolor*
AA_2840	1,3-beta-glucan synthase	0	XP_001875386	*L. bicolor*
AA_35913	1,3-beta-glucan synthase	4.39E-135	XP_001878782	*L. bicolor*
AA_3986	1,3-beta-glucan synthase	0	XP_001875386	*L. bicolor*
AA_4191	1,3-beta-glucan synthase	0	XP_001875386	*L. bicolor*
AA_3582	Beta-glucan synthesis-associated protein KRE6	0	XP_001832353	*C. cinerea*
AA_10733	Calnexin	5.4E-112	XP_001874124	*L. bicolor*
AA_10817	GTP-binding protein	0	XP_001886019	*L. bicolor*
AA_11180	GTP-binding protein	9.9E-17	XP_001875653	*L. bicolor*
AA_12377	GTP-binding protein	9.1E-50	XP_001886998	*L. bicolor*
AA_13049	GTPase-activating protein	1.8E-23	XP_001873518	*L. bicolor*
AA_1311	GTP-binding protein	1.3E-65	XP_002910724	*C. cinerea*
AA_7238	RHO GDP-GTP exchange protein	2.4E-30	XP_001831782	*C. cinerea*
AA_2919	RHO GDP-GTP exchange protein	2.2E-63	XP_001884214	*L. bicolor*
AA_8141	Rho-GTPase-activating protein	1.2E-128	XP_001880853	*L. bicolor*
AA_3923	Rho-GTPase-activating protein LRG1	0	XP_001875234	*L. bicolor*
AA_4316	ROT1 protein	3.0E-85	XP_001876159	*L. bicolor*

**Table 5:** Genes involved in steroid and terpenoid biosynthesis.

EST ID	Gene name	E-Value	Accession no.	Organism	KEGG pathway
AA_10646	Lanosterol synthase	0	XP_001883811	*L. bicolor*	S & T
AA_30963	Sterol-C4-methyl oxidase	8.6E-36	XP_001876145	*L. bicolor*	S
AA_10270	Sterol-C4-methyl oxidase	1.4E-143	XP_001838294	*C. cinerea*	S
AA_10712	Hydroxysteroid dehydrogenase	3.4E-151	XP_001836310	*C. cinerea*	S
AA_4258	Squalene synthase	6.2E-97	XP_001831087	*C. cinerea*	S & T
AA_1030	Lanosterol 14-alpha-demethylase	2.0 E-60	EGN98858	*S. lacrymans*	S
AA_15016	Lanosterol 14-alpha-demethylase	2.0E-92	XP_001836522	*C. cinerea*	S
AA_23055	Lanosterol 14-alpha-demethylase	3.0E-53	XP_001880615	*L. bicolor*	S
AA_6554	C-3 sterol dehydrogenase	5.2E-166	XP_001835665	*C. cinerea*	S
AA_24658	Hydroxymethylglutaryl-CoA reductase	5.0E-38	XP_001887308	*L. bicolor*	T
AA_10067	Phosphomevalonate kinase	1.5E-118	XP_002911641	*C. cinerea*	T
AA_7767	Diphosphomevalonate decarboxylase	0	XP_001873551	*L. bicolor*	T
AA_11136	Diphosphomevalonate decarboxylase	7.2E-33	XP_001830848	*C. cinerea*	T
AA_4584	Isopentenyl-diphosphate delta-isomerase	3.6E-153	XP_001873794	*L. bicolor*	T
AA_5415	Farnesyl diphosphate synthase	0	XP_001879398	*L. bicolor*	T

S, steroid biosynthesis; T, terpenoid biosynthesis.

### Proteome analysis of *A. aegerita*

**Figure 9:**
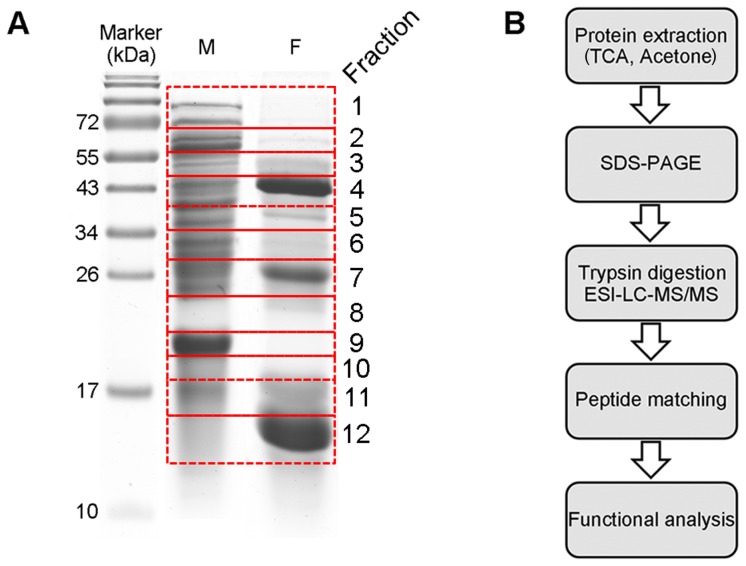
SDS-PAGE profile of samples and the experimental workflow of proteomics analysis. (A) One-dimensional SDS-PAGE separation of *A. aegerita* mycelium and fruiting body proteins. (B) The workflow of *A. aegerita* proteomics analysis.

Total proteins from mycelium and fruiting body were separated by 12.5% SDS-PAGE for 1-DGE analysis, the gels were divided into twelve sections (1–12) (**Figure 9A**). The work flow for proteomics analysis was shown in **Figure 9B**, the tryptic peptides derived from the gel bands were analyzed by LC-MS/MS, and acquired MS/MS data were searched against *A. aegerita* protein database translated from transcriptome using the MASCOT search engine. We identified a total of 309 non-redundant proteins from *A. aegerita*. Some of the noteworthy proteins were detected from *A. aegerita* proteome (**Table 6**). Ubiquitin-proteasome system, a well-known protein degradation system, is regarded as an important regulatory mechanism in cell cycle and growth [91]. Hemolytic proteins including pleurotolysin and aegerolysin were also identified by proteome analysis. Fungal hemolysins are aggregating proteins that create pores in membranes, and can lyse cells besides RBCs [92]. Furthermore, aegerolysins were reported to be specifically expressed during fruiting initiation of *A. aegerita* in previous studies [93], [94], suggesting that hemolysins played an important role in initial phase of mushroom fruiting. Some enzymes such as NADH-quinone oxidoreductase [95], copper radical oxidase [96], glycoside hydrolases and polysaccharide lyase [97] were also identified from *A. aegerita*. All the enzymes suggested the ecological importance of *A. aegerita* due to their roles in biomass degradation, and these enzymes have potential in various applications such as biodegradation of plant biomass and biofuel production.

**Table 6:** A list of proteins identified from mycelium and fruiting body.

Fraction^a^	EST^b^	protein name	Accession no.	matched peptides^c^	InterProScan	organism
2	AA_8155	Glycoside hydrolase family 38 protein	XP_001881173	1	IPR1113. Glyco_hydro-type_carb-bd	*L. bicolor*
4	AA_11009	ATP-dependent Zn protease	XP_001837465	1	IPR3593. AAA+_ATPase	*C. cinerea*
5	AA_3882	Glyceraldehyde 3-phosphate dehydrogenase	XP_001879716	1	IPR2831. GlycerAld/Erythrose_P_DH	*L. bicolor*
6	AA_4342	Glycoside hydrolase family 13 protein	XP_001877038	1	IPR647. 1-4-A-glucan_branch_enz	*L. bicolor*
7	AA_10684	polysaccharide lyase family 8 protein	XP_001873208	2	IPR8929. Chondroitin_lyas	*L. bicolor*
11	AA_30461	Aa41-PRI4	AAP49844	1	-	*A. aegerita*
12	AA_10724	Glycoside hydrolase family 88 protein	XP_001878174	2	IPR8928. 6-hairpin_glycosidase-like	*L. bicolor*
1,9	AA_4761	copper radical oxidase	XP_001838268	1	IPR1522. DUF1929	*C. cinerea*
2,3,7	AA_658	Glycoside hydrolase family 5 protein	XP_003028651	2	IPR1547. Glyco_hydro_5	*S. commune*
6	AA_7364	Glycoside hydrolase family 15 protein	XP_001884761	1	IPR8928. 6-hairpin_glycosidase-like	*L. bicolor*
7	AA_5454	glutathione-S-transferase	ACF15452	3	IPR1987. Glutathione-S-Trfase_C-like	*P. chrysosporium*
8	AA_4261	NADH:quinone oxidoreductase	XP_001831148	3	IPR8254. Flavodoxin/NO_synth	*C. cinerea*
12	AA_10071	ricin B-like lectin	AEE98238	2	IPR772. Ricin_B_lectin	*M. procera*
1,2,3,4,7	AA_11002	Metallopeptidase	XP_001835672	7	IPR2479. MetalloPept_cat_dom	*C. cinerea*
1	AA_10317	Glycoside hydrolase family 3 protein	XP_001879679	1	IPR198. Glyco_hydro_3_AS	*L. bicolor*
1,7,9,10,12	AA_3821	Aegerolysin Aa-Pri1	O42717	6	IPR9413. Aegerolysin	*A. aegerita*
3,4,5,7	AA_18943	chaperonin-60	ACR56325	4	IPR2423. Cpn6/TCP-1	*T. versicolor*
2,4,5,6,7,9	AA_24111	pleurotolysin B	BAD66667	7	IPR2864. MACPF	*P. ostreatus*
6,7,8	AA_3815	proteasome subunit	XP_001835816	7	IPR165. Proteasome_bsu_CS	*C. cinerea*
7,8	AA_8859	Mn superoxide dismutase	XP_001878774	9	IPR1189. Mn/Fe_SOD	*L. bicolor*

a : Fraction number corresponded to Fig. 9A.

b : Matched ESTs from *A. aegerita* transcriptome.

c : Peptide sequences were shown in Table S9.

### Functional annotation of non-redundant proteins

**Figure 10:**
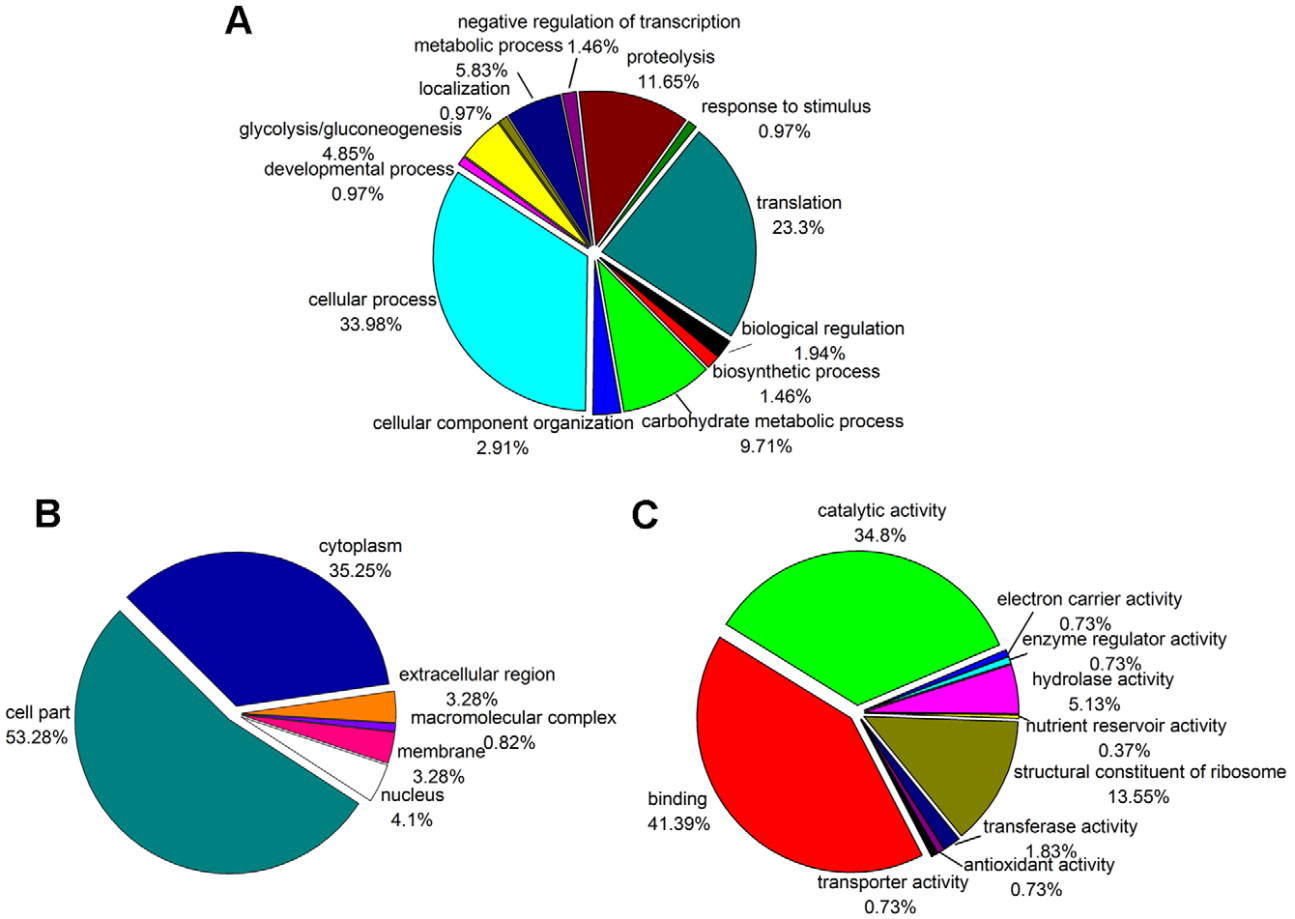
GO categories of the proteins from *A. aegerita*. The pie chart shows the distribution of the non-redundant proteins into biological process categories (A), cellular component categories (B) and molecular function categories (C) in percentage.

On the basis of annotations from NCBI and UniProt databases, all the non-redundant proteins were functionally categorized to GO terms and were represented by pie diagrams in **Figure 10**. Under the biological process category (**Figure 10A**), most of proteins were involved in “cellular process” (33.98%), “translation” (23.3%) and “proteolysis” (11.65%). Under the cellular component category (**Figure 10B**), more proteins were classified as “cell part” (53.28%) and “cytoplasm” (35.25%). Under the molecular function category (**Figure 10C**), 41.39% and 34.8% of the proteins were classified to the terms of “binding” and “catalytic activity”, respectively.

**Figure 11:**
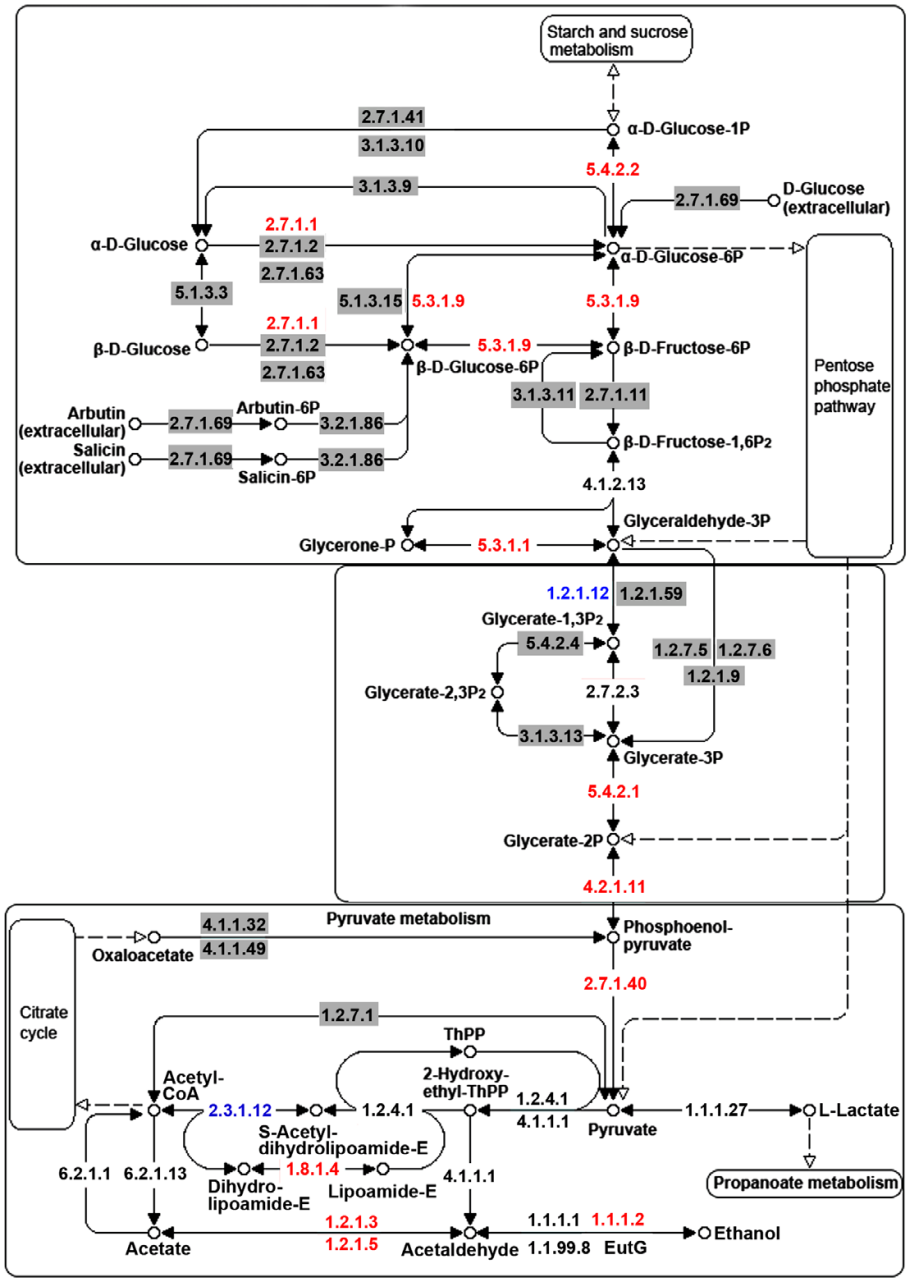
Map of carbohydrate metabolism in *A. aegerita*. Genes corresponding to enzymes shown in black or red were identified in *A. aegerita* transcriptome database, whereas those indicated in a grey shadow were not. Enzymes written in red or blue were found among proteins identified by LC-MS/MS analysis.

The majority of identified ESTs (**Figure 3**) and proteins (**Figure 10**) were involved in metabolism and included enzymes of metabolic pathways, such as glycolysis and TCA cycle (**Table S3 & S9**). Glycolysis and TCA cycles were considered to be the major routes of carbohydrate catabolism during fruiting body development in *P. ostreatus* [98] and *L. bicolor* [88]. Glycolysis is a central pathway that produces important precursor metabolites including glucose-6-phosphate and pyruvate [99]. The first step in glycolysis is phosphorylation of glucose to form glucose-6-phosphate (G-6-P), which is then rearranged into fructose-6-phosphate (F-6-P) by glucose phosphate isomerase (EC 5.3.1.9). F-6-P is phosphorylated to F-1,6-P_2_ by 6-phosphofructokinase (EC 2.7.1.11). F-1,6-P_2_ is then converted into glyceraldehyde 3-phosphate (GAP) that proceeds further into glycolysis. Subsequent enzymatic reactions result in the production of 2-phosphoglycerate (2-PG), which is converted into phosphoenol-pyruvate (PEP) by enolase (EC 4.2.1.11). Pyruvate kinase (EC 2.7.1.40) then catalyzes the conversion of PEP to pyruvate (**Figure 11**). The putative components involved in glycolysis were identified from the *A. aegerita* transcriptome and proteome databases based on sequence homology. The results demonstrated that most of the enzymes involved in glycolysis were identified by LC-MS/MS peptide analysis, and genes encoding the enzymes required for almost all the steps in this pathway were represented in the *A. aegerita* transcriptome database (**Figure 11**). The integration of high-throughput sequencing and LC-MS/MS protein profiling is an effective strategy to establish deep transcriptome and proteome databases for the investigation of metabolic pathways in *A. aegerita*. Further studies are needed to get a deeper understanding of the complicated metabolic pathways underlying *A. aegerita* development.

## Conclusions

This study is the first exploration to characterize the transcriptome and proteome of *A. aegerita* using high-throughput sequencing and liquid chromatography tandem MS. In the current study, a list of transcripts and proteins were identified and categorized to provide a valuable resource for further study of this mushroom. We also conducted gene expression analysis to explore metabolic pathways of bioactive components in *A. aegerita*. The results indicated that steroid biosynthesis was more active in mycelium while polysaccharide biosynthesis was up-regulated in fruiting body. Moreover, the integration of *A. aegerita* transcriptome and proteome revealed a number of enzymes that potentially catalyzed steps in carbohydrate metabolism. These important resources increase the potential application of this species in nutrition, human health, and biomass degradation.

## Materials and Methods

### Sample preparation

Mycelia were cultured on a potato dextrose agar (PDA) medium by incubating at 28°C in the dark (90% air humidity). After 14 days, the mycelium fully covered the petri dish (diameter = 9 cm) and fruiting process was induced from vegetative cultures in accordance with a previous procedure [100]. Samples from the two development stages (mycelium and fruiting body) were harvested and frozen at −80°C until RNA extraction.

### cDNA library construction and sequencing

The total RNA from mycelium and fruiting body was isolated using TriZol reagent (Promega) according to the manufacturer's instruction. RNA quantity and quality were checked using a NanoDrop 1000 spectrophotometer (NanoDrop Technologies) and an Agilent 2100 Bioanalyzer (Agilent Technologies, Santa Clara, CA) and the two samples had RNA Integrity Number (RIN) value more than 8.5. A total of 20 µg RNA was equally pooled from the two samples for cDNA library. The samples for transcriptome analysis were prepared by using an Illumina kit by following manufacturer's recommendations. Briefly, mRNA was concentrated from total RNA using oligo (dT) magnetic beads and then fragmented using divalent cations. The cleaved RNA fragments were used for first strand cDNA synthesis using random primers and reverse transcriptase. This was followed by second strand cDNA synthesis using DNA polymerase I and RNaseH. These cDNA fragments then went through an end repair process and ligation of adapters. Following agarose gel electrophoresis and extraction of cDNA from gels, the cDNA fragments with the lengths of 200 bp (±25 bp) were purified and enriched by PCR to construct the final cDNA libraries.

The two cDNA libraries were sequenced at Beijing Genome Institute (BGI, Shenzhen, China) on the Illumina sequencing platform (GAIIx). The fluorescent images process to sequences, base-calling and quality value calculation were performed by the Illumina data processing pipeline, and sequence.txt files (in FASTQ format) were generated. A total of 14 million reads with the lengths of 75 bp were obtained from one plate (8 lanes) in a single sequencing run, generating approximately 2.13 gigabase pairs (Gbp) of raw data (**Table S1**).

### De novo assembly of sequencing reads

The work flow for transcriptome annotation was shown in **Figure S1**. Prior to assembly, the 75-bp raw reads were filtered by removing adaptor sequences, duplication sequences, ambiguous reads, and low-quality reads using a quality cut-off value of 40. *De novo* assembly of the clean reads was performed using SOAPdenovo program [101] which implements a de Bruijn graph algorithm. First, short reads were assembled into longer but gapless contigs. Then reads were mapped back to contigs, unknown sequences between each pair of contigs were replaced with ‘N’s, and scaffolds were produced. Paired-end reads were used again for gap filling of scaffolds to obtain expressed sequences tags (ESTs) with least ‘N’s, which cannot be extended on either end. Finally, the overlapping ESTs from each sample were further spliced and assembled to yield the maximum length non-redundant ESTs using sequence clustering software-TGI Clustering tools [102] and Cap3 [103]. The final EST set was representative of sequences with least ‘N’s, and was used for further analysis in this study.

### Homology searches and function annotation

Functional annotation of *A. aegerita* transcriptome was performed by running our assembly against the NCBI nr, COG (http://www.ncbi.nlm.nih.gov/COG) and KEGG (http://www.genome.jp/kegg/) databases using BLASTx (E-value<10^−5^). Homology searches were carried out by query of the NCBI non-redundant protein database using BLASTx algorithm (E-value<10^−5^) [104]. Gene names were assigned to each assembled sequence based on the best BLAST hit. The blast results were then imported into Blast2GO program [105] for mapping the sequences into Gene Ontology (GO) terms. We then used WEGO software [106] to analyze the GO functional classification for the ESTs and to understand the distribution of gene functions of *A. aegerita* from the macro level. ESTs were also aligned to the COG database [107] to predict and classify potential functions based on known orthologous sequences. KEGG database [108] was used for studying complex metabolic pathways in *A. aegerita*.

### Differential expression analysis

The expression level of each EST was estimated by the frequency of clean reads in the corresponding sample. We used RPKM method (Reads Per kb per Million reads) for the calculation of read density. By taking into account the variations of gene length and the total mapped number of sequencing reads, the RPKM measure gives normalized values of gene expression, which enabled transcript comparisons between samples [51]. Briefly, we mapped back the filtered reads to various assembled ESTs, estimated total mapped reads, uniquely mapped reads assigned to each assembled EST, with maximum two mismatches allowed. According to the RPKM method, gene expression levels were calculated with the following formula: RPKM (A) = (1,000,000×*C*×1,000)/(*N*×*L*) (Assigns RPKM (A) to be the expression of gene A, *C* to be number of reads that uniquely aligned to gene A, *N* to be total number of reads that uniquely aligned to all genes, and *L* to be the number of bases on gene A).

In this study, the Illumina sequencing reads were mapped to the ESTs using SOAPaligner (http://soap.genomics.org.cn/soapaligner.html) [109]. Then we applied the R package DEGseq [110] to identify differentially expressed ESTs with the random sampling model based on the read count for each EST at different developmental stages. FDR (false discovery rate) [111] was used to determine the threshold of p value in multiple tests. We used the absolute value of log_2_Ratio>1 and FDR≤0.001 as the threshold to determine the differentially expressed ESTs between the two developmental stages [52]. GO and KEGG annotations were applied to describe the function of differentially expressed ESTs as stated above (E<10^−5^).

### Expression profiling by semi-quantitative RT-PCR

The differential expression of a selection of 7 genes identified as being differentially expressed was validated by applying semi-quantitative RT-PCR. Total RNAs from mycelium and fruiting body of *A. aegerita* were isolated using TriZol reagent (Promega) and were treated with RNase-free DNase I (TaKaRa), and about 2 µg of total RNA of each sample was reverse-transcribed by M-MLV reverse transcriptase (Promega) using oligo (dT) as primer. The PCR was carried out using the following thermal cycling profile: 95°C for 5 min, followed by 34 cycles of amplification (95°C for 30 sec, 58°C for 30 sec, and 72°C for 45 sec), and 72°C for 10 min. The sequences of the primer pairs designed using software Premier 5.0 were listed in **Table S2**. The PCR products and their sizes were examined using 1% agarose gel electrophoresis. *Actin* gene of *A. aegerita* was amplified as an endogenous loading control for testing the validity of template preparation. The expression of each gene was confirmed in at least three rounds of independent RT-PCR reactions.

### Extraction of total protein

Total protein extraction was carried out using the trichloroacetic acid/acetone (TCA/acetone) extraction protocol [12]. Frozen mycelium and fruiting body (1 g) were ground to a fine powder in liquid nitrogen using pre-chilled ceramic mortar and pestle, transferred to a pre-chilled centrifuge tube. The powdered extract from each sample was used for proteomics analysis. Proteins were extracted from tissue powder (200 mg) by addition of 1.6 mL ice-cold acetone containing 10% (w/v) TCA and 2% (v/v) 2-mercaptoethanol (2-ME). The mixture was homogenized by inverting the tube 10 times, and proteins were precipitated for 1 h at −20°C. The suspension was centrifuged at 15,000 rpm for 15 min at 4°C. The supernatant was discarded, and the pellet was washed three times with chilled wash buffer (0.07% 2-ME, 2 mM EDTA, and EDTA-free proteinase inhibitor cocktail tablets (Roche) in 100% acetone), followed by removal of all the acetone. Pellets were dried under vacuum and stored at −80°C until used.

### One-Dimensional Gel Electrophoresis and Mass Spectrometry Analysis

Proteins were solubilized in homogenization buffer [0.2 M Tris-HCl buffer, pH 7.8, containing 5 mM EDTA•2Na, 14 mM 2-ME, 10% (v/v) glycerol, and 2 EDTA-free proteinase inhibitor tablets (Roche) per 100 mL of buffer solution in MQ H_2_O]. To effectively solubilize the protein pellet, sodium dodecyl sulfate (SDS)-sample buffer [2.5×, 62 mM Tris (pH 6.8) containing 10% (v/v) glycerol, 2.5% (w/v) SDS, and 5% (v/v) 2-ME, pH 6.8 was added to the mixture, followed by vortexing and sonication (water bath). The mixture was centrifuged at 15,000 rpm for 15 min at 4°C. The supernatant was used for protein quantification by BCA (bicinchoninic acid) protein assay kit. Before electrophoresis, a drop of bromophenol blue (BPB) was added to the protein samples and the mixture boiled for 5 min at 95°C. 100 µg of protein from each sample was resolved on a 12.5% SDS-PAGE gel. The gel was stained with Coomassie brilliant blue (CBB) R-250, and divided into 12 sections (**Figure 9A**). The proteins were digested by trypsin at 37°C for 18 h allowing to one miscleavage. The tryptic peptides derived from the gel bands were separated by C-18 reverse-phase column and analyzed on a nanoelectrospray ionization mass spectrometer (nESI-LC-MS/MS) operated in the positive ion mode. The samples were loaded onto precolumn and washed with the loading solvent (0.1% formic acid in H_2_O; flow rate, 4 µl/min) for 10 min to remove salts. Subsequently, a Switchos II column switching device transferred flow paths to the analytical column. The nanoflow eluted at a flow rate of 400 nl/min using a 90 min gradient elution from 90% solvent A to 90% solvent B, where solvent A was distilled water and solvent B was acetonitrile containing 0.1% (v/v) formic acid. And the column outlet was coupled directly to the high voltage ESI source, which was interfaced to the amaZon ETD mass spectrometer (Bruker, Germany). The nanospray voltage was typically 1.5 kV in the nESI-LC-MS/MS mode. The nESI-LC-MS/MS was acquired in ‘Information Dependent Acquisition’ mode, which allows the user to acquire MS/MS spectra based on an inclusion mass list and dynamic assessment of relative ion intensity. The data acquisition time was set to 3 s per spectrum over m/z range of 300–1500 Da. The MS/MS data were further processed with Flexanalysis (Bruker Daltonics, http://www.bdal.de) with peak picking parameters recommended for ion trap data.

The generated peak-lists were searched against *A. aegerita* protein database translated from transcriptome using the MASCOT software package (Version 2.1, Matrix Sciences, U.K.; www.matrixscience.com). Search parameters were set to 0.6 Da and 1.0 Da for peptide and fragment mass tolerance, respectively. Only tryptic cleavages were considered and one missed cleavage was permitted. The cut-off score, determined by Mascot using a 0.05 significance threshold (p<0.05), was 40. To find the exact proteins and the functions for these proteins, the sequences identified from the tryptic digests were analyzed by using BLASTp (E≤10^−5^) against NCBI fungal protein database. The proteome was functionally classified based on the annotations from online search against UniProt database (http://www.uniprot.org/uniprot/). All the peptides identified by LC-MS/MS analysis were shown in **Table S9**.

### Data deposition

The raw Illumina sequencing data of *A. aegerita* were submitted to NCBI Sequence Read Archive (SRA, http://www.ncbi.nlm.nih.gov/Traces/sra) under the accession number of SRA026731. The *de novo* assembly sequence data were deposited in the NCBI's Transcriptome Shotgun Assembly (TSA) database with the accession numbers JW839591–JW861025.

## Supporting Information

Figure S1 Flowgram representing data processing pipeline for *de novo* transcriptome assembly and annotation of *A. aegerita*.
(TIF)

Figure S2 The top 25 KEGG categories of *L. bicolor* (A) and *S. commune* (B).
(TIF)

Figure S3 KEGG reference pathway map of purine metabolism. Components identified in *A. aegerita* transcriptome are framed in red.
(TIF)

Figure S4 KEGG reference pathway map of starch and sucrose metabolism. Components identified in *A. aegerita* transcriptome are framed in red.
(TIF)

Figure S5 ESTs that were differentially expressed in mycelium and fruiting body transcriptomes.
(TIF)

Table S1 Throughput and quality of Illumina sequencing of *A. aegerita* transcriptome.
(DOC)

Table S2 Primers sequences used for RT-PCR validation of Illumina sequencing data.
(DOC)

Table S3 Sequences with top BLAST hits against NCBI-nr database. All *A. aegerita* ESTs were aligned against the NCBI-nr database using BLASTx with a cutoff E-value of 10^−5^.
(XLS)

Table S4 ESTs encoding NRPSs and PKSs in *A. aegerita*.
(DOC)

Table S5 Cytochrome P450 families identified in *A. aegerita* transcriptome.
(XLS)

Table S6 Differentially expressed ESTs at each of the two developmental stages. RPKM, Reads Per kb per Million. False discovery rate was set at FDR≤0.001. The ESTs were compared with sequences in nr database using BLASTx (cut-off E-value = 10^−5^).
(XLS)

Table S7 The ten most differentially expressed ESTs at the two developmental stages.
(DOC)

Table S8 Genes involved in TCA cycle.
(DOC)

Table S9 List of peptides from mycelium and fruiting body identified by LC-MS/MS analysis.
(XLS)
