# Supplementary figures and images for: Transcriptome and Proteome Exploration to Provide a Resource for the Study of Agrocybe aegerita
Source: PLoS One. 2013 Feb 13;8(2):e56686. doi: 10.1371/journal.pone.0056686 (PMC3572045; doi:10.1371/journal.pone.0056686)

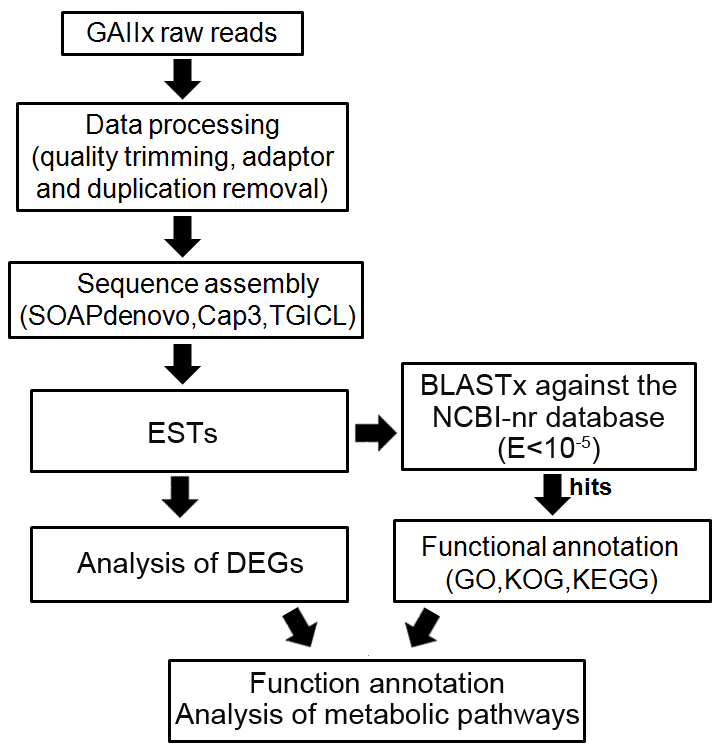

Supplement: Figure S1 — Flowgram representing data processing pipeline for de novo transcriptome assembly and annotation of A. aegerita. (TIF) [file pone.0056686.s001.tif]

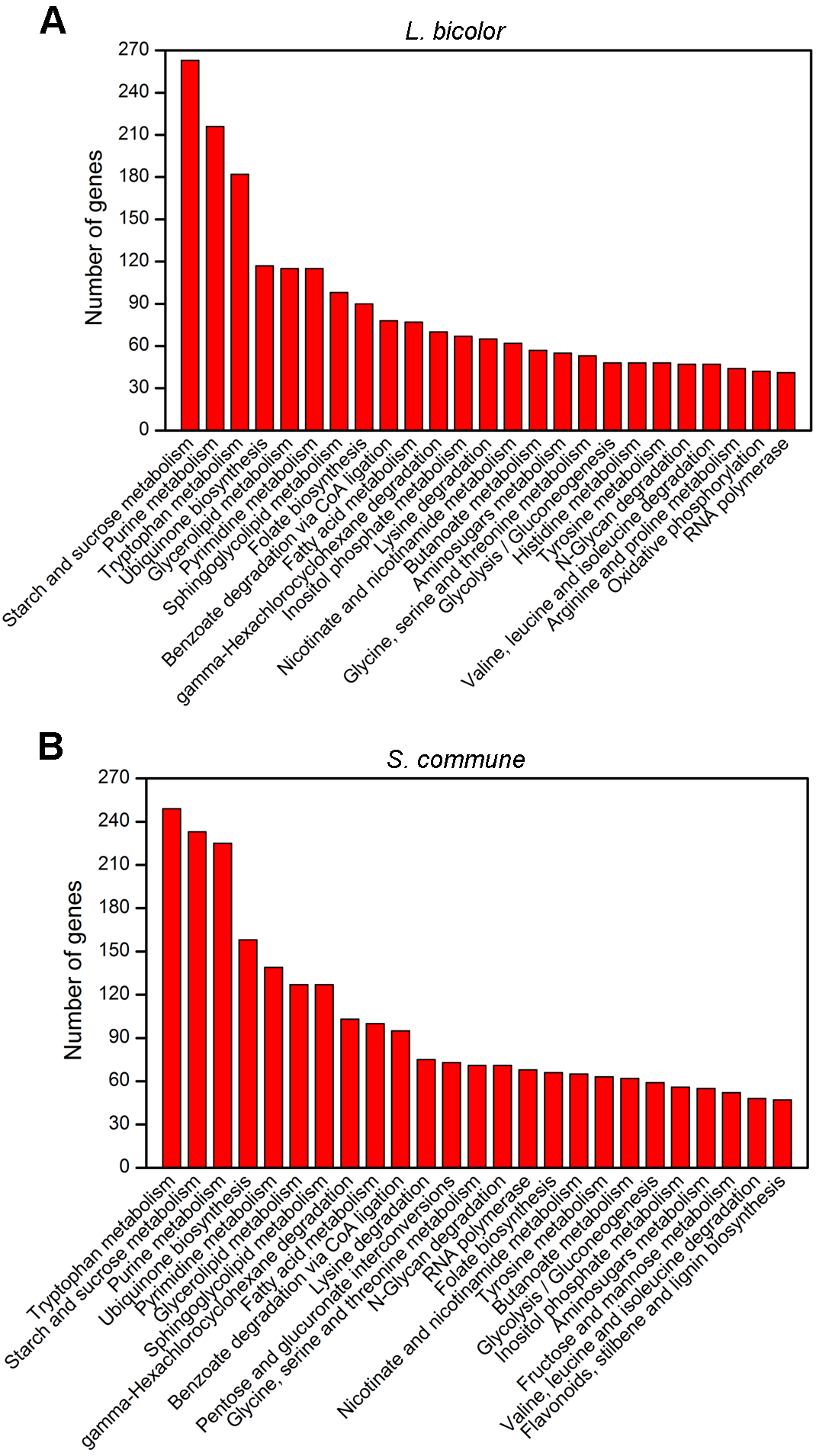

Supplement: Figure S2 — The top 25 KEGG categories of L. bicolor (A) and S. commune (B). (TIF) [file pone.0056686.s002.tif]

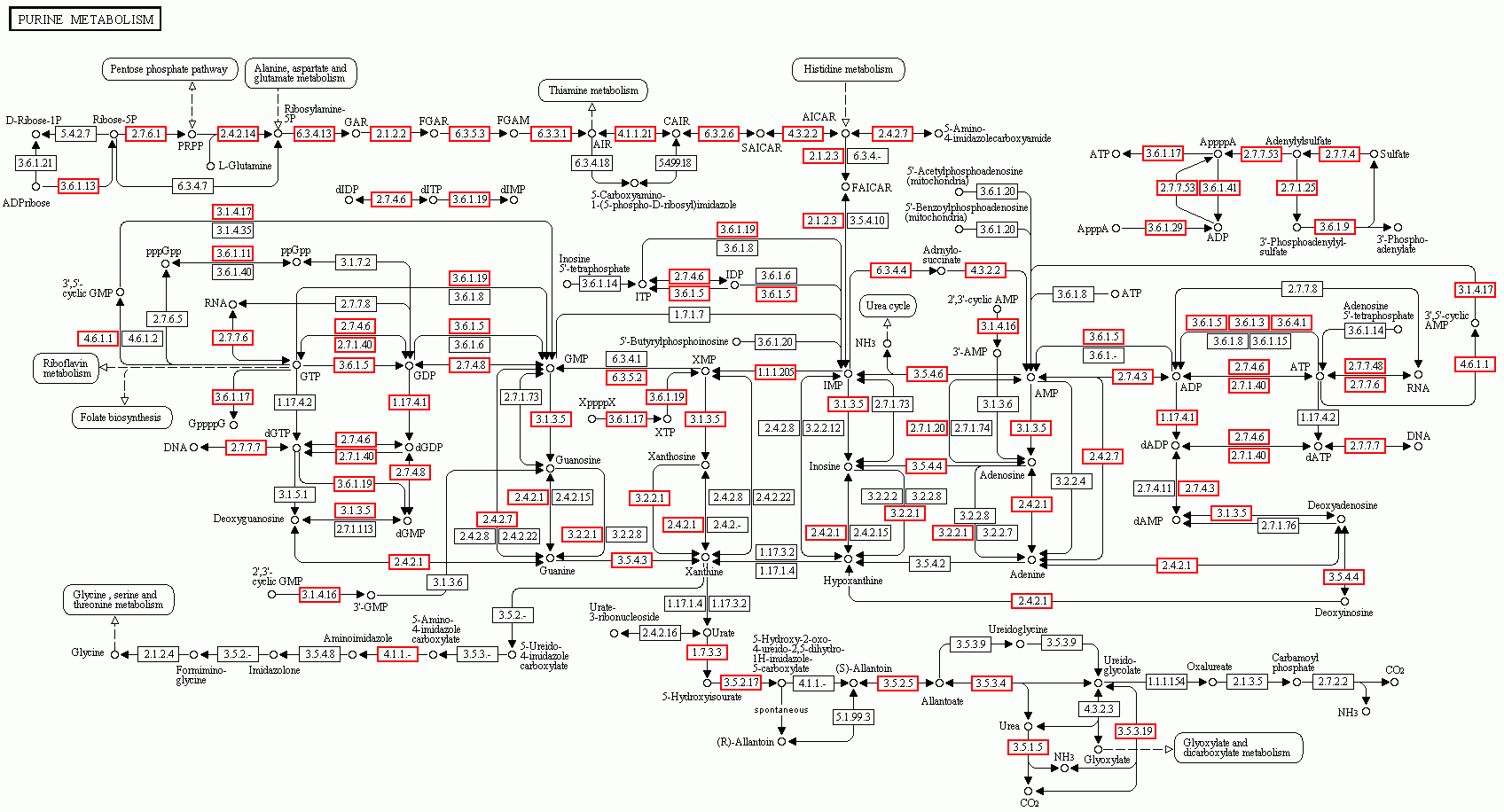

Supplement: Figure S3 — KEGG reference pathway map of purine metabolism. Components identified in A. aegerita transcriptome are framed in red. (TIF) [file pone.0056686.s003.tif]

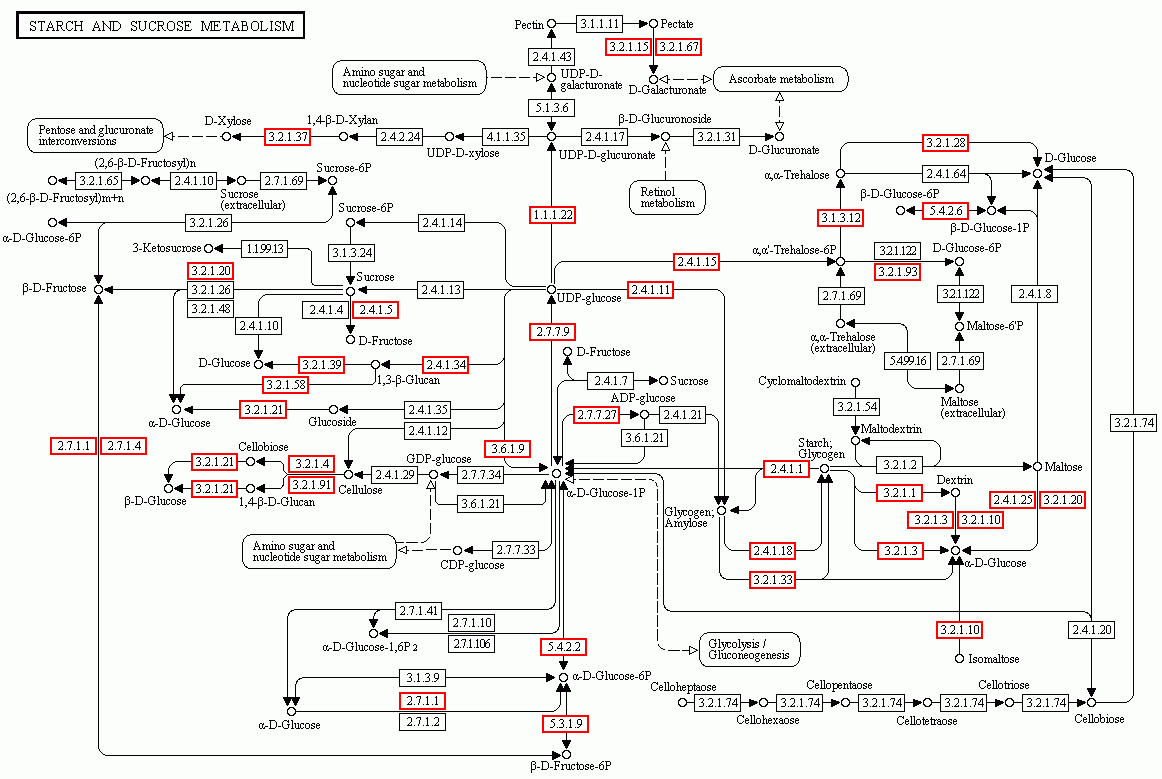

Supplement: Figure S4 — KEGG reference pathway map of starch and sucrose metabolism. Components identified in A. aegerita transcriptome are framed in red. (TIF) [file pone.0056686.s004.tif]

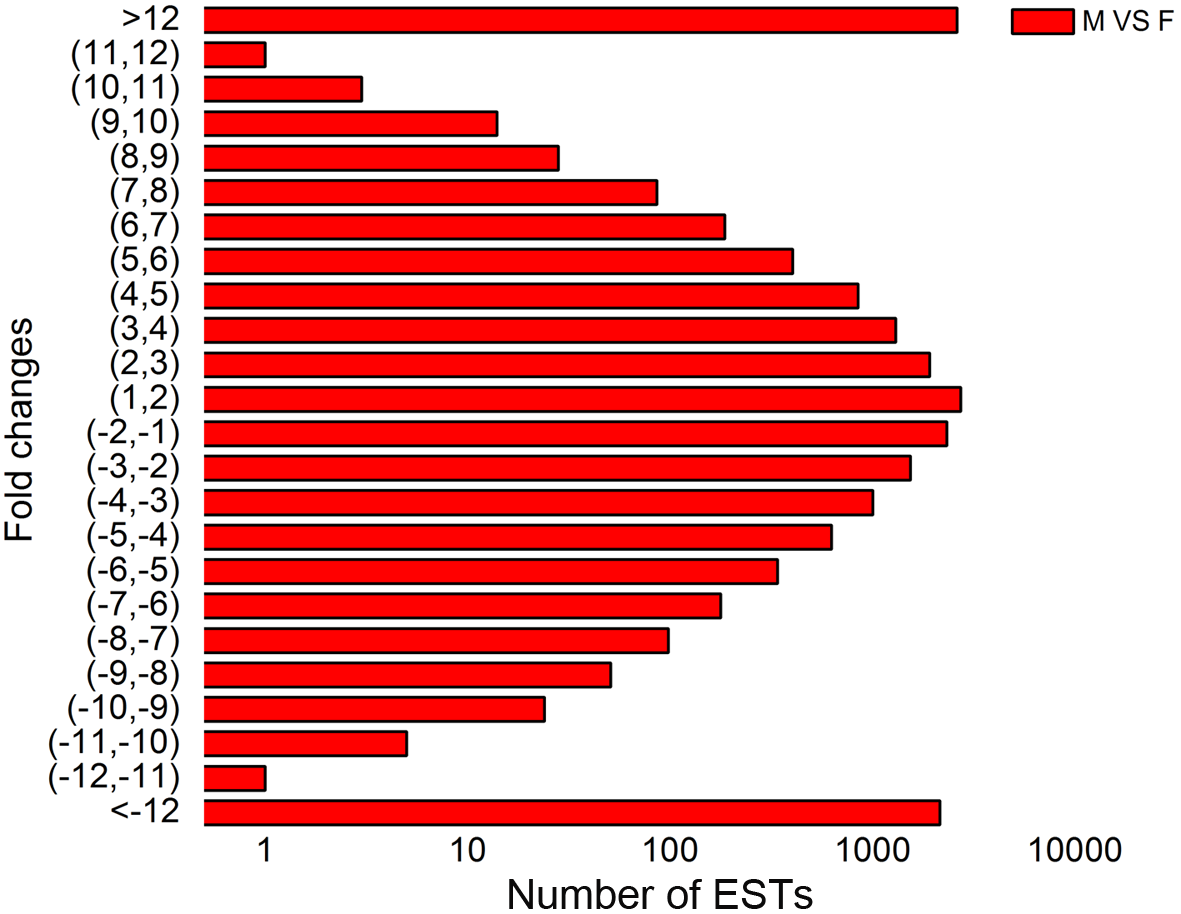

Supplement: Figure S5 — ESTs that were differentially expressed in mycelium and fruiting body transcriptomes. (TIF) [file pone.0056686.s005.tif]
