# Supplementary material for: Transcriptome and Proteome Exploration to Provide a Resource for the Study of Agrocybe aegerita
Source: PLoS One. 2013 Feb 13;8(2):e56686. doi: 10.1371/journal.pone.0056686 (PMC3572045; doi:10.1371/journal.pone.0056686)
Supplement: Table S1 — Throughput and quality of Illumina sequencing of A. aegerita transcriptome. (DOC) [file pone.0056686.s006.doc]

**Table S1.** Throughput and quality of Illumina sequencing of *A. aegerita* transcriptome.

| Samples | Total Reads | Total Nucleotides (nt) | Q20 percentage^a^ | N percentage^b^ | GC percentage |
| --- | --- | --- | --- | --- | --- |
| mycelium | 7.55 M | 1116 M | 98.75% | 0.03% | 52.80% |
| fruiting body | 6.86 M | 1014 M | 98.69% | 0.03% | 52.67% |

a, Q20 percentage indicates the percentage of sequences with sequencing error rate lower than 1%.

b, N percentage indicates the percentage of nucleotides could not be sequenced.
