# Supplementary material for: Transcriptome and Proteome Exploration to Provide a Resource for the Study of Agrocybe aegerita
Source: PLoS One. 2013 Feb 13;8(2):e56686. doi: 10.1371/journal.pone.0056686 (PMC3572045; doi:10.1371/journal.pone.0056686)
Supplement: Table S2 — Primers sequences used for RT-PCR validation of Illumina sequencing data. (DOC) [file pone.0056686.s007.doc]

**Table S2.** Primers sequences used for RT-PCR validation of Illumina sequencing data.

|  |  |  |
| --- | --- | --- |
| **Targeted genes** | **Length of ESTs** | **Sequence of the primer pairs** |
| *AA_19720* | 158 bp | 5'-cacattgaagtaccccatcg-3' |
|  |  | 5'-aaacatgatctgggtcatct-3' |
| *AA_10787* | 282 bp | 5'-acactatcgctacaggttcg-3' |
|  |  | 5'-tgtccaggtgcggattcttc-3' |
| *AA_10799* | 200 bp | 5'-cctcgctatcctctatgaag-3' |
|  |  | 5'-ggaaaggggtagtaatgagg-3' |
| *AA_33178* | 174 bp | 5'-tcttccaccgctcccacctt-3' |
|  |  | 5'-acgaccagtccgagaagagc-3' |
| *AA_34533* | 221 bp | 5'-aaccactcaaacctcaccca-3' |
|  |  | 5'-gccaacgacgacaccaacaa-3' |
| *AA_34539* | 280 bp | 5'-gcgttgtcgtccaggatgtt-3' |
|  |  | 5'-cgataaagcgtcgggttcat-3' |
| *AA_36106* | 202 bp | 5'-gtcttatgctccccactaca-3' |
|  |  | 5'-ccacttgcctactgctccga-3' |
| *AA_12497* | 226 bp | 5'-tacctcctccacatcgcctt-3' |
|  |  | 5'-tcagacccgagatttgcttc-3' |
