# Supplementary material for: Transcriptome and Proteome Exploration to Provide a Resource for the Study of Agrocybe aegerita
Source: PLoS One. 2013 Feb 13;8(2):e56686. doi: 10.1371/journal.pone.0056686 (PMC3572045; doi:10.1371/journal.pone.0056686)
Supplement: Table S4 — ESTs encoding NRPSs and PKSs in A. aegerita. (DOC) [file pone.0056686.s009.doc]

**Table S4.** ESTs encoding NRPSs and PKSs in *A. aegerita*.

| EST ID | Type | Accession number | Organism | E-value |
| --- | --- | --- | --- | --- |
| AA_4113 | NRPS | EGN93241 | *S. lacrymans* | 5.84E-20 |
| AA_492 | NRPS | EGO25609 | *S. lacrymans* | 4.39E-101 |
| AA_9351 | NRPS | EGN94460 | *S. lacrymans* | 2.91E-25 |
| AA_1078 | NRPS | XP_001835921 | *C. cinerea* | 0 |
| AA_13127 | NRPS | EGO25609 | *S. lacrymans* | 4.88E-38 |
| AA_17133 | NRPS | EGO25609 | *S. lacrymans* | 7.49E-91 |
| AA_17811 | NRPS | XP_001836663 | *C. cinerea* | 1.24E-116 |
| AA_22796 | NRPS | ABA62019 | *L. edodes* | 3.61E-34 |
| AA_22924 | NRPS | ABA62019 | *L. edodes* | 4.35E-15 |
| AA_2379 | NRPS | XP_003026223 | *S. commune* | 3.62E-70 |
| AA_24659 | NRPS | XP_001835921 | *C. cinerea* | 1.53E-58 |
| AA_27927 | NRPS | XP_001836648 | *C. cinerea* | 1.58E-22 |
| AA_10787 | PKS | EIN06933 | *P. strigosozonata* | 0 |
| AA_28490 | PKS | XP_001833231 | *C. cinerea* | 3.50E-57 |
| AA_35325 | PKS | XP_001833231 | *C. cinerea* | 6.22E-24 |
| AA_9190 | PKS | XP_001833231 | *C. cinerea* | 2.50E-11 |
| AA_14072 | PKS | XP_001833231 | *C. cinerea* | 7.00E-25 |
| AA_14330 | PKS | XP_001833231 | *C. cinerea* | 8.21E-38 |
| AA_15860 | PKS | XP_001833231 | *C. cinerea* | 9.41E-21 |
| AA_17266 | PKS | EGN93241 | *S. lacrymans* | 1.41E-27 |
| AA_22561 | PKS | XP_001833231 | *C. cinerea* | 5.60E-43 |
| AA_25172 | PKS | XP_001833231 | *C. cinerea* | 5.09E-58 |
| AA_27319 | PKS | EGO18627 | *S. lacrymans* | 7.19E-68 |
