# Supplementary material for: Transcriptome and Proteome Exploration to Provide a Resource for the Study of Agrocybe aegerita
Source: PLoS One. 2013 Feb 13;8(2):e56686. doi: 10.1371/journal.pone.0056686 (PMC3572045; doi:10.1371/journal.pone.0056686)
Supplement: Table S7 — The ten most differentially expressed ESTs at the two developmental stages. (DOC) [file pone.0056686.s012.doc]

**Table S7.** The ten most differentially expressed ESTs at the two developmental stages.

| **EST ID** | **Length (bp)** | **log_2_Ratio (M/F)** | **Regulation mode (M/F)** | **P-value** | **FDR** | **Gene description** |
| --- | --- | --- | --- | --- | --- | --- |
| AA_21574 | 251 | 27.12 | up | 0 | 0 | Aa1-Pri4 |
| AA_33085 | 218 | -26.70 | down | 0 | 0 | peptidase 1 |
| AA_35373 | 395 | -26.56 | down | 0 | 0 | methyl-accepting chemotaxis protein |
| AA_34441 | 287 | -26.23 | down | 0 | 0 | hypothetical protein |
| AA_35425 | 409 | -26.22 | down | 0 | 0 | transcriptional regulator |
| AA_20018 | 203 | 25.97 | up | 0 | 0 | predicted protein |
| AA_20037 | 204 | 25.95 | up | 0 | 0 | predicted protein |
| AA_20020 | 203 | 25.91 | up | 0 | 0 | predicted protein |
| AA_33927 | 258 | -25.79 | down | 0 | 0 | predicted protein |
| AA_23924 | 473 | 25.76 | up | 0 | 0 | predicted protein |

M, mycelium; F, fruiting body; FDR, false discovery rate.
