# Supplementary material for: Transcriptome and Proteome Exploration to Provide a Resource for the Study of Agrocybe aegerita
Source: PLoS One. 2013 Feb 13;8(2):e56686. doi: 10.1371/journal.pone.0056686 (PMC3572045; doi:10.1371/journal.pone.0056686)
Supplement: Table S8 — Genes involved in TCA cycle. (DOC) [file pone.0056686.s013.doc]

| **EST ID** | **Gene name** | **E-value** | **Accession number** | **Organism** |
| --- | --- | --- | --- | --- |
| AA_34399 | citrate synthase | 5.60E-52 | XP_001838767 | *C. cinerea* |
| AA_3497 | citrate synthase | 0 | XP_001880764 | *L. bicolor* |
| AA_10600 | citrate synthase | 0 | XP_001880983 | *L. bicolor* |
| AA_10676 | citrate synthase | 1.26E-94 | XP_001874823 | *L. bicolor* |
| AA_1174 | citrate synthase | 9.67E-152 | XP_001833371 | *C. cinerea* |
| AA_15963 | aconitate hydratase | 6.03E-52 | XP_002912138 | *C. cinerea* |
| AA_27131 | aconitate hydratase | 2.62E-158 | EGN96504 | *S. lacrymans* |
| AA_30583 | aconitate hydratase | 2.34E-58 | XP_001873889 | *L. bicolor* |
| AA_4668 | aconitate hydratase | 0 | XP_001834931 | *C. cinerea* |
| AA_10811 | isocitrate dehydrogenase | 0 | XP_001876704 | *L. bicolor* |
| AA_12727 | isocitrate dehydrogenase | 5.78E-121 | XP_002394333 | *M. perniciosa* |
| AA_14489 | isocitrate dehydrogenase | 1.10E-61 | XP_001877144 | *L. bicolor* |
| AA_36127 | isocitrate dehydrogenase | 0 | XP_001876714 | *L. bicolor* |
| AA_9201 | 2-oxoglutarate dehydrogenase | 0 | XP_001876081 | *L. bicolor* |
| AA_9803 | succinate dehydrogenase | 1.67E-77 | XP_001878561 | *L. bicolor* |
| AA_12475 | succinate dehydrogenase | 3.03E-18 | XP_001878561 | *L. bicolor* |
| AA_12476 | succinate dehydrogenase | 3.60E-18 | XP_001878561 | *L. bicolor* |
| AA_9084 | succinate dehydrogenase | 3.09E-13 | EGN99401 | *S. lacrymans* |
| AA_9119 | malate dehydrogenase | 4.09E-52 | XP_001881221 | *L. bicolor* |
| AA_9499 | malate dehydrogenase | 5.22E-61 | XP_001839443 | *C. cinerea* |
| AA_10324 | malate dehydrogenase | 1.06E-118 | XP_001834158 | *C. cinerea* |
| AA_2136 | malate dehydrogenase | 0 | XP_001832332 | *C. cinerea* |
| AA_24428 | malate dehydrogenase | 1.82E-156 | XP_001835695 | *C. cinerea* |
| AA_30173 | malate dehydrogenase | 3.27E-88 | XP_001832332 | *C. cinerea* |
| AA_33499 | malate dehydrogenase | 3.89E-21 | BAH80448 | *L. edodes* |
| AA_33538 | malate dehydrogenase | 1.05E-37 | XP_002393196 | *M. perniciosa* |
| AA_34462 | malate dehydrogenase | 4.19E-26 | XP_001881221 | *L. bicolor* |
| AA_7131 | malate dehydrogenase | 0 | XP_001875905 | *L. bicolor* |

**Table S8.** Genes involved in TCA cycle.
